# Statin use in cancer survivors versus the general population: cohort study using primary care data from the UK clinical practice research datalink

**DOI:** 10.1186/s12885-018-4947-8

**Published:** 2018-10-22

**Authors:** Kendal Chidwick, Helen Strongman, Anthony Matthews, Susannah Stanway, Alexander R. Lyon, Liam Smeeth, Krishnan Bhaskaran

**Affiliations:** 1 London School of Hygiene and Tropical Medicine, London, UK; 2 Department of Non-Communicable Diseases Epidemiology, London School of Hygiene and Tropical Medicine, Keppel Street, London, WC1E 7HT UK; 3 Breast Unit, Royal Marsden Hospital, London, UK; 4 Imperial College London, London, UK

**Keywords:** Cancer survivor, Cardiovascular risk, Statin, Persistence, Primary care

## Abstract

**Background:**

Cancer survivors may be at increased risk of cardiovascular diseases, but little is known about whether prescribing guidelines for the primary prevention of cardiovascular disease are adequately implemented in these patients. We compared levels of statin initiation and cessation among cancer survivors compared to the general population to determine differences in uptake of pharmaceutical cardiovascular risk prevention measures in these groups.

**Methods:**

The study population included individuals aged ≥40 during 2005–13 within the UK Clinical Practice Research Datalink primary care database. Within this population we identified cancer survivors who were alive and under follow-up at least 1 year after diagnosis, and controls with no cancer history. Follow-up time prior to cancer diagnosis was included in the control cohort. Using logistic regression, we compared these groups with respect to uptake of statins within 1 month of a first high recorded cardiovascular risk score. Then, we used Cox modelling to compare persistence on statin therapy (time to statin cessation) between cancer survivors and controls from the main study population who had initiated on a statin.

**Results:**

Among 4202 cancer survivors and 113,035 controls with a record indicating a high cardiovascular risk score, 23.0% and 23.5% respectively initiated a statin within 1 month (adjusted odds ratio 0.98 [91.8–1.05], *p* = 0.626). Cancer survivors appeared more likely to discontinue statin treatment than controls (adjusted hazard ratio 1.07 [1.01–1.12], *p* = 0.02). This greater risk of discontinuing was only evident after the first year of therapy (p-interaction < 0.001).

**Interpretation:**

Although cardiovascular risk is thought to be higher in cancer survivors compared to the general population, cancer survivors were no more likely to receive statins, and marginally more likely to cease long-term therapy, than general population controls. There may be an opportunity to mitigate the suspected higher cardiovascular risk in the growing population of cancer survivors by improving uptake of lipid-lowering treatment and persistence on therapy.

**Electronic supplementary material:**

The online version of this article (10.1186/s12885-018-4947-8) contains supplementary material, which is available to authorized users.

## Introduction

Advances in cancer detection and treatment over the past 20 years have led to considerable improvements in cancer survival [1, 2]. Consequently, the number of people living with a history of cancer – “cancer survivors” - is steadily increasing [1]. Some cancers are increasingly thought of as chronic diseases that require evidence based multidisciplinary care for the prevention and management of comorbidities and sequelae [3–6].

Recent evidence has demonstrated that cancer survivors are at increased risk of cardiovascular disease (CVD) compared to the general population [7] and that in certain populations (e.g. breast cancer survivors) CVD competes with cancer as the leading cause of death [8, 9]. This is likely to be due to shared risk factors (e.g. obesity and tobacco use) and potential cardiotoxic effects of systemic therapies and radiotherapy [10, 11]. It has been suggested that sub-optimal primary prevention may also be responsible [12, 13]. In the absence of specific cardiovascular risk prevention guidelines targeted at cancer survivors, such individuals would be expected to follow strategies recommended for the general population, i.e. reducing lifestyle-related risk factors, and pharmacological management of lipid and blood pressure [14]. Since 2005, UK guidelines on primary CVD prevention (Additional file 1: Table S1) have recommended all people with high cardiovascular risk (i.e. 10 year predicted risk ≥20%) be offered statin therapy [15]. Current risk assessment tools do not take possible additional risks associated with previous cancer into account, and may under-estimate vascular risk among cancer survivors. Statin uptake rates in the general population appear to be low, with a number of studies finding that only around one fifth to one third of patients at high cardiovascular risk were actually prescribed a statin [16–19]. To our knowledge, equivalent statin uptake rates in cancer survivors have not previously been quantified.

In order to best target initiatives to improve gaps in survivorship care, we need to confirm whether cardiovascular risk prevention strategies are being followed in this vulnerable population, and whether cancer survivors persist with long-term medications intended to reduce their cardiovascular risk. We therefore aimed to compare use of lipid-lowering drugs between cancer survivors and control patients with no previous cancer, specifically focusing on prescribing of statins to those scored as high CV risk; and the time to statin discontinuation, among those starting a statin.

## Methods

### Data source and main study population

We conducted a longitudinal population-based open cohort study using Clinical Practice Research Datalink (CPRD) GOLD data. CPRD GOLD is a primary care database containing the anonymised medical records for over 15 million patients from 674 general practices in the United Kingdom (UK) [20]. CPRD GOLD data, which are collated daily from participating practices that use Vision software, include information on demographics, symptoms, tests, diagnoses, and prescriptions, and are subject to data quality checks. Diagnoses in CPRD are typically recorded using National Health Service (NHS) Read codes. Approximately 7% of the UK population are included, with patients broadly representative of the UK general population in terms of age, sex and ethnicity [20]. In the UK, the General Practitioner (GP) is the first point of contact and “gatekeeper” for most non-emergency NHS care; the vast majority of the population is registered with a GP [21].

The study period was 1st January 2005 to 31st December 2013 because during this period national guidelines (Additional file 1: Table S1) remained constant in recommending statin therapy for people with predicted cardiovascular risk ≥20% over 10 years, for primary prevention. Patients who were 40 years or older during the study period and had at least 12 months of research quality CPRD follow-up prior to entering the cohort were included; follow-up began on the latest of 1/1/2005, the patient’s 40th birthday, or the date on which 12 months of follow-up had been accumulated in CPRD; follow-up ended on the earliest of 31/12/2013, the last data collection date, or the end of follow-up in CPRD. Patients with a CVD event (Additional file 2: Table S2) prior to entering the study were excluded (as our focus was on primary CVD prevention).

Read code lists to identify cancer diagnoses were developed using the methodology described in a previous study [22]. All person-time was categorised into “never-cancer”, “first year post-cancer diagnosis” and “≥ 1 year post-cancer diagnosis”. Person-time in the ≥1 year post-cancer diagnosis category made up the cancer survivor group, while “never-cancer” person-time made up the control group. Person-time in the first year post-cancer diagnosis was not analysed further. People could contribute to more than one of the comparison groups, if they developed cancer during follow-up, but could only contribute to one exposure group at any given moment of follow-up. From this overall study population, sub-cohorts were defined to address each of our objectives, as described in the sections below.

### Descriptive analysis

Baseline characteristics were described for the overall study population and sub-populations (defined below). We also described the proportion of patients with at least one measurement recorded in the past 5 years for each of: blood pressure (diastolic and systolic recorded on the same day), lipids (total cholesterol and/or high-density lipoprotein cholesterol (HDL) and/or cholesterol/HDL ratio), and 10 year predicted cardiovascular risk score (Framingham, QRisk, ASSIGN, Joint British Society (JBS), or unspecified; Read terms used to identify cardiovascular risk scores are included in Additional file 3: Table S3). This description of blood pressure/lipids/risk score recording was stratified by cancer survivor / control status, gender and specific points of age (namely at age 45, and in steps of 5 years up to 75); and within strata, was restricted to individuals under follow-up at the given age and with at least 5 years of prior follow-up in CPRD.

### Uptake of statins among those recorded as high CVD risk

To assess uptake of statin therapy among patients with high recorded CVD risk, all patients from the overall study population with a first ever Read code or quantitative record indicating predicted 10 year cardiovascular risk of ≥20% in CPRD and no previous statin use were included in the ‘high cardiovascular risk cohort’. To exclude patients with previous high risk scores, patients were required to have at least 12 months of research quality follow-up in CPRD prior to the first high risk score record. Patients were excluded if their follow-up ended during the 31 day period after the high cardiovascular risk score, leaving inadequate eligible time to assess prescription of a statin within 1 month. For this analysis, patients were grouped as cancer survivors or controls based on their status on the date of their first high CVD risk record. The outcome was statin uptake, defined as having a first prescription for statin therapy (defined using the codes in Additional file 4: Table S4) within 31 days of the first high cardiovascular risk score. The association between cancer survivorship and statin uptake was assessed using logistic regression. First, the unadjusted association was estimated, then we adjusted for age at baseline (i.e. at first high cardiovascular risk score, in 10 year categories) and gender as a-priori confounders. The following potential confounders were considered one at a time: practice Index of Multiple Deprivation quintile (a proxy for socio-economic status) [23]; body mass index (BMI, categorised as underweight (< 18.5 kg/m^2^), healthy weight (18.5-25 kg/m^2^) and overweight/obese (> 25 kg/m^2^)); total cholesterol (categorised as normal (< 5 mmol), moderate-high (5-8 mmol) and high (> 8 mmol)); smoking status (never, current, ex-smoker); alcohol consumption (non-drinker, ex-drinker, high/medium/low/unknown-level current drinker); chronic kidney disease (CKD), diabetes, and chronic liver disease at baseline (both defined using Read codes); and calendar time (split into 2005–07, 2008–10, 2011–13 to account for the marked increase in statin prescribing demonstrated in previous CPRD research over the study time period [24]). These covariates were only included in the final model if the absolute change to the odds ratio (OR) was greater than 10% when the single variable was added to the unadjusted model. Only participants with complete data for the relevant confounder were included in the stepwise testing analyses (adjusted and comparative a-priori models).

We investigated whether calendar period modified the association between cancer and initiation of a statin by adding calendar period to the model as an interaction term - the stratified ORs were assessed and a likelihood ratio test was used to compare the models with and without the interaction term. There was not considered to be any a priori reason to test for any other interactions.

### Time to statin discontinuation among those starting a statin

To assess statin persistence, patients from the overall study population who started a statin for primary prevention (i.e. with no previous CVD record) during the study time period were included in the ‘statin initiator cohort’. To exclude prevalent statin users, patients were required to have at least 12 months of research quality follow-up prior to first prescription. For this analysis, patients were grouped as cancer survivors or controls based on their status on the date of their first statin prescription. The outcome was the time from statin initiation to first cessation of therapy, calculated from prescription records. The end date of each prescription was calculated by dividing the quantity prescribed by the daily dose where this information was available. Where duration could not be calculated, the modal prescription duration (28 days) was assumed. If there was no repeat prescription within 90 days after the estimated end date of a prescription, a patient was defined as having ceased therapy at the estimated end date. A multivariate Cox model was built, with time since statin initiation as the underlying time scale and with age (time-updated), gender, BMI, smoking status and diabetes (time updated) included as a priori confounders. All regression analyses were restricted to individuals with complete data for these confounders. Other candidate confounders were assessed and added to the model using the same model-building strategy as described for the analysis of statin uptake (above).

We investigated an interaction between cancer survivorship and calendar period as uptake of statins varies over time and drivers of this may differ in cancer survivors versus the general population. We also considered heterogeneity by time since cancer diagnosis. The proportional hazards assumption was assessed by testing for an interaction between cancer survivor status and time since statin initiation (categorised as < 0.5, 0.5–1, > 1–2, > 2 years). In a sensitivity analysis, the Cox model time scale was switched to age (to control more closely for age), and time since initiation was adjusted for as a time-varying covariate. Robust standard errors were used in all analyses to account for clustering at GP practice level.

## Results

**Fig. 1:**
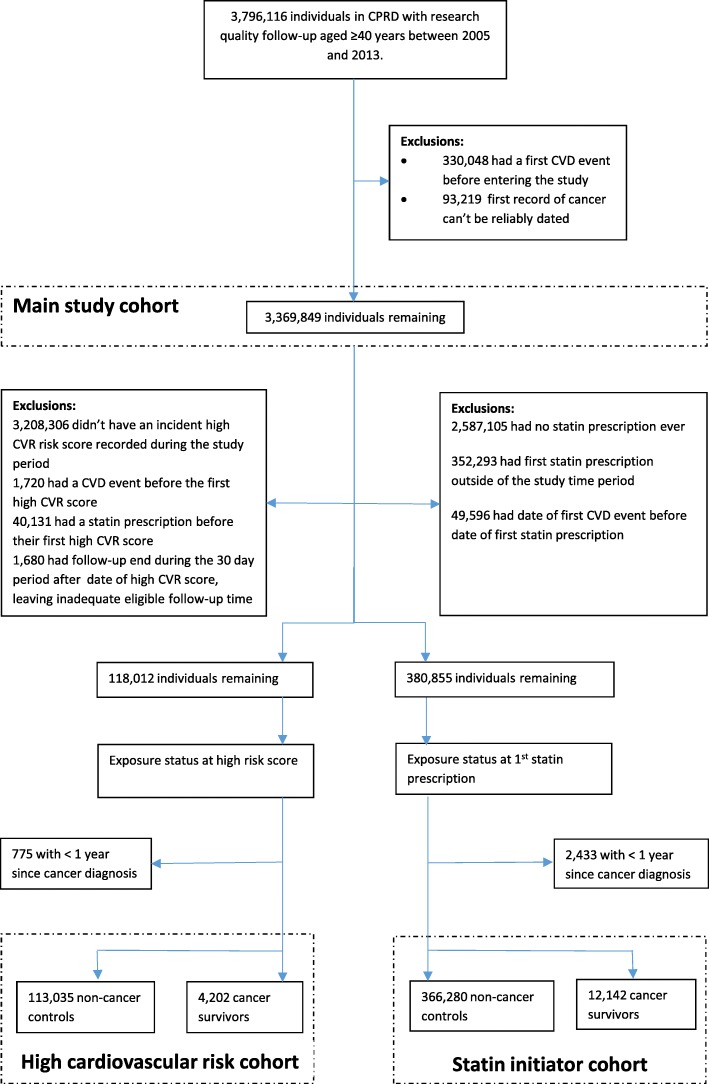
Flow chart of included study participants

Overall, 3,369,849 individuals aged 40 years or over during the study period 2005 to 2013 were included in the main study cohort (Fig. 1). Of the main study cohort 131,676 contributed person-time to the cancer survivors (exposed) and 3,324,152 to the general population controls (unexposed). Demographics and baseline characteristics of the study populations are described in Table 1.

**Table 1:** Baseline characteristics for the main study cohort and sub-populations by cancer survivor (> = 1 year) status

	Main Study Cohort^a^		High Cardiovascular Risk Cohort^b^		Statin Initiator Cohort^c^	
	Cancer Survivor	Control	Cancer Survivor	Control	Cancer Survivor	Control
	*n* = 131,676	*n* = 3,324,152	*n* = 4202	*n* = 113,035	*n* = 12,142	*n* = 366,280
	*n* (%)	*n* (%)	*n* (%)	*n* (%)	*n* (%)	*n* (%)
Sex						
Male	58,587 (44.7)	1,618,820 (48.70)	2659 (63.3)	80,264 (71.0)	5959 (49.1)	194,430 (53.1)
Female	72,823 (55.3)	1,705,299 (51.30)	1543 (36.7)	32,771 (29.0)	6183 (50.9)	171,848 (46.9)
Age (years)						
Median (IQR)	66.5 (57.5–75.5)	50.5 (42.1–62.3)	69.1 (64.2–72.9)	64.2 (58.4–69.8)	68.9 (62.4–75.1)	62.6 (54.9–70.2)
40–49	14,117 (10.7)	1,529,272 (46.0)	27 (0.6)	5410 (4.8)	351 (2.9)	45,132 (12.3)
50–59	24,564 (18.6)	775,972 (23.3)	368 (8.8)	27,121 (24.0)	1630 (13.4)	96,664 (26.7)
60–69	38,078 (28.9)	521,777 (15.7)	1815 (43.2)	50,847 (45.0)	4393 (36.2)	125,540 (34.3)
70–79	33,983 (25.8)	303,231 (9.1)	1765 (42.0)	26,713 (23.6)	4086 (33.7)	73,598 (20.1)
80+	20,934 (15.9)	193,900 (5.8)	227 (5.4)	2944 (2.6)	1682 (13.7)	25,346 (6.9)
BMI (kg/m2)						
Mean (SD)	26.9 (5.3)	26.9 (5.3)	28.3 (4.8)	27.8 (5.1)	28.2 (5.4)	28.7 (5.6)
< 18.5	2748 (2.1)	48,109 (1.4)	50 (1.2)	897 (0.8)	131 (1.1)	2906 (0.8)
18.5–25	46,037 (35.0)	1,130,838 (34.0)	1127 (26.8)	27,294 (24.2)	3175 (26.9)	86,962 (24.5)
≥ 25	74,375 (56.5)	1,736,648 (52.2)	2925 (69.6)	81,734 (72.3)	8504 (72.0)	265,128 (74.7)
Missing	8516 (6.5)	408,557 (12.3)	100 (2.4)	3113 (2.8)	332 (2.7)	11,285 (3.1)
Cholesterol (mmol/L)						
Mean (SD)	5.3 (1.2)	5.4 (1.1)	5.7 (1.0)	5.8 (1.1)	6.0 (1.3)	6.1 (1.3)
Normal (< 5)	42,503 (32.3)	796,230 (23.9)	898 (21.4)	21,543 (19.1)	2242 (18.7)	58,780 (16.2)
Mod (5–8)	62,529 (47.5)	1,405,997 (42.3)	3137 (74.6)	85,221 (75.4)	8965 (74.8)	278,848 (76.9)
High (> 8)	1704 (1.5)	38,297 (1.2)	92 (2.2)	3316 (2.9)	775 (6.47)	25,032 (6.9)
Missing	24,940 (18.9)	1,083,578 (32.6)	75 (1.8)	2955 (2.6)	157 (1.29)	3640 (1.0)
Smoking status						
Never	46,606 (35.4)	1,358,706 (40.9)	1194 (28.4)	29,306 (25.9)	3729 (30.7)	118,728 (32.4)
Ex	61,969 (47.1)	965,510 (29.1)	2133 (50.8)	46,494 (41.2)	6572 (54.1)	166,482 (45.5)
Current	22,627 (17.2)	885,915 (26.6)	875 (20.8)	37,190 (32.9)	1840 (15.2)	80,841 (22.1)
Missing	474 (0.4)	114,021 (3.4)	< 5	45 (0.0)	< 5	299 (0.1)
Alcohol status (non- ex- current drinker)						
Non	25,030 (19.0)	577,211 (17.4)	742 (17.7)	18,139 (16.1)	2500 (27.3)	75,516 (27.5)
Ex	3778 (2.84)	74,249 (2.2)	162 (3.9)	4079 (3.6)	447 (4.9)	13,002 (4.7)
Current						
light	48,354 (36.7)	1,042,681 (31.3)	1629 (38.8)	40,996 (36.3)	4868 (53.1)	140,039 (51.0)
moderate	4776 (3.6)	106,934 (3.2)	224 (5.3)	5904 (5.2)	503 (5.5)	15,837 (5.8)
heavy	5025 (3.8)	150,372 (4.5)	254 (6.0)	9229 (8.2)	562 (6.1)	19,668 (7.2)
unknown	2819 (2.1)	74,769 (2.3)	117 (2.8)	4193 (3.7)	280 (3.1)	10,747 (3.9)
Missing	41,894 (31.8)	1,297,936 (39.0)	1074 (25.6)	30,495 (27.0)	2982 (24.6)	91,474 (25.0)
Medical history						
Diabetes	12,045 (9.2)	153,405 (4.6)	187 (4.5)	5308 (4.7)	2239 (18.4)	69,156 (18.9)
CKD	9577 (7.3)	28,419 (0.9)	368 (8.8)	5465 (4.8)	1450 (12.0)	24,464 (6.7)
Chronic liver disease	2223 (1.7)	39,429 (1.2)	75 (1.8)	7975 (1.8)	221 (1.8)	6378 (1.7)
Index of Multiple Deprivation quintile						
1 (least)	16,993 (12.9)	298,566 (9.0)	641 (15.2)	12,842 (11.4)	1697 (11.1)	40,458 (11.1)
2	12,043 (12.9)	340,205 (10.2)	588 (14.0)	13,616 (12.6)	1607 (13.2)	43,074 (11.8)
3	18,708 (14.2)	384,484 (11.6)	545 (13.0)	14,211 (12.6)	1655 (13.6)	48,491 (13.2)
4	64,007 (48.6)	1,978,653 (59.5)	1894 (45.1)	57,464 (50.8)	5615 (46.2)	188,142 (51.4)
5 (most)	14,985 (11.4)	322,244 (9.7)	434,534 (12.7)	14,902 (13.2)	1586 (13.1)	46,115 (12.6)
Calendar year						
2005–07	62,916 (47.8)	2,423,116 (72.9)	623 (14.8)	23,020 (20.4)	4105 (33.8)	156,589 (42.8)
2008–10	34,416 (26.1)	471,289 (14.2)	1681 (40.0)	48,349 (42.8)	4310 (35.5)	124,339 (33.9)
2011–13	34,344 (26.1)	429,747 (12.9)	1898 (45.2)	41,666 (36.9)	3727 (30.7)	85,352 (23.3)

^a^Covariates were measured at baseline (or closest recording) defined in the ‘main study cohort’ as the date of the start of unexposed (non-cancer) and exposed (≥ 1 year post cancer diagnosis) follow-up time. Patients in the Main Study Cohort could contribute person-time to exposed and unexposed cohorts

^b^Baseline was defined in the ‘high cardiovascular risk cohort’ as the date of the first high cardiovascular risk score. Patients could only be included in one of the comparison cohorts

^c^Baseline was defined in the ‘statin initiator cohort’ as the date of the first statin prescription. Patients could only be included one of the comparison cohorts. If a non-cancer patient developed cancer while on statin therapy they were censored

**Fig. 2:**
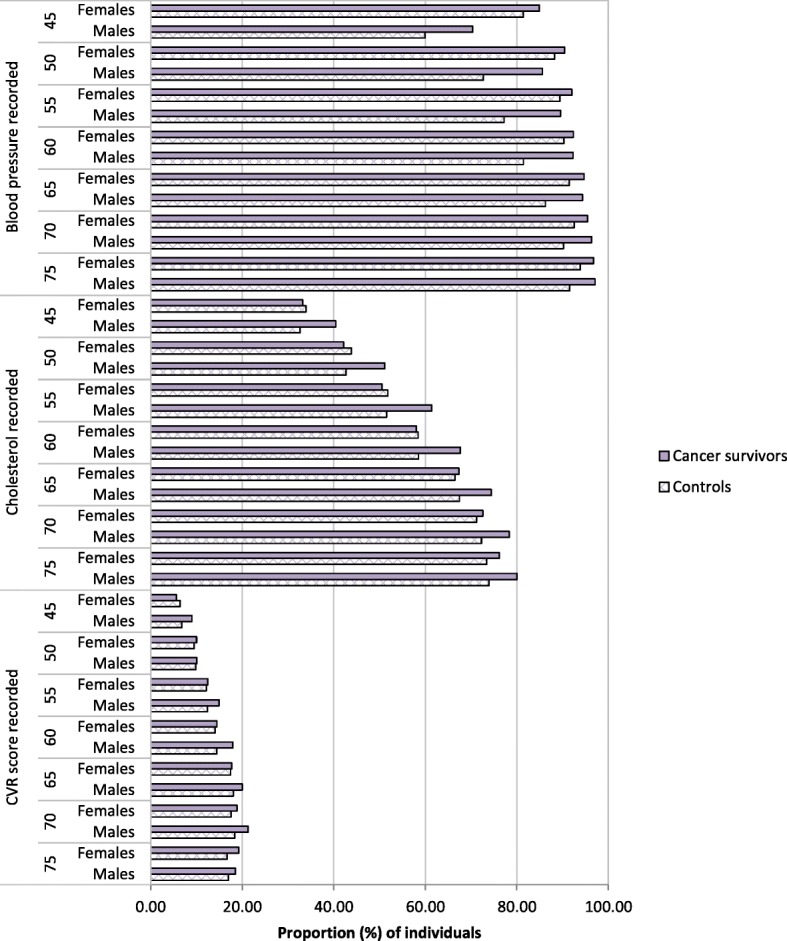
Proportion (%) of individuals with a cardiovascular risk measures recorded in the past 5 years. • Blood pressure recorded = a diastolic and systolic BP recorded on the same day at least once in the past 5 years. • Cholesterol recorded = total cholesterol and/or high-density lipoprotein cholesterol and/or cholesterol/HDL ratio recorded at least once in the past 5 years. • Cardiovascular risk score (CVR) recorded = 10 year predicted cardiovascular risk (Framingham, QRisk, ASSIGN, Joint British Society (JBS), or unspecified) recorded at least once in the past 5 years. • Study population = 3,369,849 patient main study cohort

Cancer survivors were older on average than controls (median: 67 years vs. 51 years), and a higher proportion were females (55.3% vs. 51.3%). As patients enter the cancer survivor group later than the general population control group, the proportion of patients followed up from later calendar years was greater in the cancer survivor group (26.1% vs. 12.9% for 2011–2013). The baseline prevalence of diabetes and CKD were substantially higher among cancer survivors compared with controls (9.2% vs 4.6% respectively for diabetes, and 7.3% vs 0.9% for CKD). Figure 2 describes the proportion of individuals from the full study population with a BP, cholesterol measurement, or a cardiovascular risk score recorded at least once in the past 5 years, by age, gender and exposure status; a table of the same results presented numerically is provided in Additional file 5: Table S5. Overall, the highest level of monitoring was observed for BP (range: 60.0% to 97.2%), followed by cholesterol (range: 32.6% to 80.1%). BP and cholesterol monitoring were consistently higher in cancer survivors than controls, across all ages and gender with the exception of cholesterol monitoring in women which appeared to be similar between cancer survivors and controls. There was suboptimal recording of cardiovascular risk scores for both sexes and in all age groups, regardless of cancer status (range: 5.6% to 21.3%). Cancer survivors tended to have either similar or marginally better recording of cardiovascular risk compared to controls of the same age and gender. We repeated these analyses post-hoc restricting to patients without CKD or diabetes, in case baseline differences in these diseases affected the comparison between cancer survivors and controls. Similar patterns were seen.

### Uptake of statins among those recorded as high CVD risk

The ‘high cardiovascular risk cohort’ included 118,012 individuals (Fig. 1). Of these, 4202 individuals were cancer survivors at the time of their first high risk score and 113,035 were controls; cancer survivors were more likely than controls to be older (median age 69.1 vs 64.2 years), female (36.7% vs. 29.0%), current non-smokers (79.2% vs. 67.1%), less deprived (29.2% vs 24.0% in the lower two deprivation quintiles) and to have CKD (8.8% vs. 4.8%; Table 1, middle columns). There were also small but statistically significant differences in cholesterol and alcohol status between the groups.

Nine hundred sixty-eight of 4202 cancer survivors (23.0%) and 26,606 of 113,035 controls (23.5%) initiated a statin within a month of their first high cardiovascular risk score. There was no evidence of association between cancer survivorship and statin initiation in the initial unadjusted analysis: OR 0.97 (95% CI: 0.90–1.05, *p* = 0.443, Table 2). There was evidence that statin initiation was more likely among females, those with BMI ≥25 kg/m^2^, total cholesterol > 5 mmol/L, ex-smokers, and those with diabetes or CKD, and less common in people aged over 80 years, heavy drinkers, underweight individuals, and those in later calendar periods. None of the tested covariates individually changed the association between cancer survivorship and statin initiation by more than 10%; the maximum change being 1.5%. Therefore only age and gender were included in the final logistic regression model as a priori confounders associated with both cancer status and statin initiation. The final adjusted odds ratio was 0.98 (95% CI: 0.92–1.05, *P* = 0.626) (Table 2). There was no evidence that the association between cancer survivorship and statin initiation differed by calendar period (p-interaction = 0.709).

**Table 2:** Associations between cancer survivorship and covariates, and statin initiation among the high cardiovascular risk cohort

	Total n	Initiates statin n (%)	Crude OR (95% CI)	*p*-value	Adjusted^a^ OR (95% CI)	*p*-value
Cancer status (binary)						
Non-cancer	113,035	26,606 (23.54)	1	0.443	1	
Cancer survivor	4202	968 (23.04)	0.97 (0.90–1.05)		0.98 (0.92–1.05)	0.626
Sex						
Males	82,923	18,902 (22.79)	1	< 0.001	1	< 0.001
Females	34,314	8672 (25.27)	1.15 (1.11–1.18)		1.19 (1.15–1.23)	
Age (years)						
40–49	5437	1301 (23.93)	1	< 0.001	1	< 0.001
50–59	27,489	6402 (23.29)	0.97 (0.90–1.04)		0.96 (0.89–1.03)	
60–69	52,662	12,760 (24.23)	1.02 (0.92–1.12)		1.00 (0.90–1.10)	
70–79	28,478	6624 (23.26)	0.96 (0.87–1.06)		0.92 (0.83–1.01)	
80+	3171	487 (15.36)	0.58 (0.47–0.71)		0.53 (0.43–0.65)	
BMI (kg/m2)						
< 18.5	948	136 (14.35)	0.62 (0.53–0.73)	< 0.001		
18.5–25	28,421	6050 (21.29)	1			
≥ 25	84,659	20,779 (24.54)	1.2 (1.17–1.24)			
Missing n (%)	3213 (2.74)					
Cholesterol (mmol/L)						
Normal (< 5)	22,469	2910 (12.95)	1	< 0.001		
Moderate - High (5–8)	88,274	22,838 (25.87)	2.36 (2.25–2.49)			
High (> 8)	3435	1750 (50.95)	7.1 (6.48–7.78)			
Missing n (%)	3059 (2.61)					
Smoking status						
Never smoker	30,500	6863 (22.5)	1	< 0.001		
Ex-smoker	48,627	11,971 (24.62)	1.12 (1.08–1.17)			
Current smoker	38,065	8738 (22.96)	1.03 (0.97–1.09)			
Missing n (%)	45 (0.04)					
Alcohol status						
Non-drinker	18,881	4598 (24.35)	1	< 0.001		
Ex-drinker	4241	1064 (25.09)	1.04 (0.94–1.15)			
Current drinker						
light	42,625	10,455 (24.53)	1.01 (0.96–1.06)			
moderate	6128	1475 (24.07)	0.98 (0.91–1.06)			
heavy	9483	2039 (21.50)	0.85 (0.77–0.94)			
amount unknown	4310	1009 (23.41)	0.95 (0.85–1.06)			
Missing n (%)	31,569 (26.93)					
Medical history						
Diabetes	5495	1853 (33.72)	1.7 (1.57–1.85)	< 0.001		
Chronic kidney disease	5833	1568 (26.88)	1.21 (1.11–1.31)	< 0.001		
Chronic liver disease	2050	453 (22.10)	0.92 (0.83–1.02)	0.120		
Index of Multiple Deprivation quintile						
1 (least deprived)	13,483	3133 (23.24)	1	< 0.001		
2	14,204	3143 (22.13)	0.94 (0.75–1.17)			
3	14,756	3510 (23.79)	1.03 (0.82–1.29)			
4	59,358	13,626 (22.96)	0.98 (0.84–1.16)			
5 (most deprived)	15,436	4162 (26.96)	1.22 (0.95–1.56)			
Calendar year						
2005–07	23,643	6257 (26.46)	1	< 0.001		
2008–10	50,030	11,461 (22.91)	0.83 (0.80–0.86)			
2011–13	43,564	9856 (22.62)	0.81 (0.78–0.84)			

^a^Adjusted for a priori confounders (age and gender). No other covariates changed the association between cancer survivorship and statin initiation by more than 10%

### Persistence on statin therapy for primary prevention of CVD

Three hundred eighty thousand eight hundred fifty-five individuals were newly prescribed a statin for primary prevention (Fig. 1). Twelve thousand one hundred forty-two were cancer survivors at the time of statin initiation, 366,280 were controls and 2433 were excluded from the analysis as statins were initiated within 1 year of cancer diagnosis. Characteristics of the two groups are described in Table 1 (right hand columns); differences between the groups were similar to those observed in the high cardiovascular risk cohort. In the statin survivor and control group, 6141 (50.6%) and 190,044 (51.9%) individuals discontinued statin therapy over a median follow-up time of 1.56 and 1.65 years respectively. Following exclusion of subjects with missing a-priori confounder values, 366,771 subjects (96.9%) were included in the Cox regression models. The overall discontinuation rates were 20.7 and 19.8 per 100 person years for the cancer survivor and general population groups. The unadjusted hazard ratio (HR) for association between cancer survivorship and statin discontinuation was 1.02 (95%CI: 0.96–1.09, *p* = 0.50; Table 3). Associations between covariates and statin discontinuation are also shown in Table 3.

**Table 3:** Associations between cancer survivorship covariates, and time to statin cessation among statin initiators

	Statin initiators		Number of events^a^	Person-Years of Follow-Up (hundreds)	Crude Rate (per 100 Person-Years)	Unadjusted HR^b^	*p*-value	Adjusted HR^c^	*p*-value
	n	%							
Cancer status (binary)									
Non-cancer	354,961	96.8	183,844	92.5	19.8	1	0.502	1	0.02
Cancer survivor	11,810	3.2	5962	2.8	20.7	1.02 (0.96–1.09)		1.07 (1.01–1.12)	
Sex									
Males	193,646	52.8	98,246	50.6	19.4	1	< 0.001	1	< 0.001
Females	173,125	47.2	91,560	44.7	20.5	1.05 (1.04–1.06)		1.05 (1.04–1.06)	
Age (yrs)									
40–49	44,088	12.0	26,453	9.8	27.1	1	< 0.001	1	< 0.001
50–59	95,539	26.1	51,099	24.5	20.8	0.81 (0.80–0.82)		0.80 (0.79–0.82)	
60–69	126,570	34.5	60,763	34.9	17.4	0.69 (0.68–0.70)		0.66 (0.65–0.67)	
70–79	75,425	20.6	37,647	20.5	18.4	0.73 (0.71–0.75)		0.68 (0.67–0.70)	
80+	25,149	6.9	13,844	5.7	24.2	0.89 (0.85–0.93)		1.14 (1.05–1.24)	
BMI (kg/m2)									
< 18.5	3035	0.8	1773	0.6	28.9	1.25 (1.20–1.30)	< 0.001	1.18 (1.14–1.23)	< 0.001
18.5–25	90,123	24.6	48,251	22.6	21.3	1		1	
≥ 25	273,613	74.6	139,782	72.1	19.4	0.92 (0.91–0.93)		0.94 (0.93–0.95)	
Cholesterol (mmol/L)									
Normal (< 5)	59,402	16.2	29,003	15.8	18.4	1	< 0.001		
Mod-High (5–8)	279,538	76.2	146,489	72.4	20.2	1.10 (1.09–1.11)			
High (> 8)	24,778	6.8	12,533	6.8	18.5	1.01 (0.99–1.03)			
Missing	3053	0.8							
Smoking status									
Never smoker	117,658	32.1	61,055	31.1	19.6	1	< 0.001	1	< 0.001
Ex-smoker	169,393	46.2	84,172	45.3	18.6	0.94 (0.93–0.96)		0.98 (0.96–0.99)	
Current smoker	79,720	21.7	44,579	19.0	23.5	1.16 (1.14–1.17)		1.13 (1.12–1.15)	
Alcohol status									
Non-drinker	76,637	20.9	40,349	20.0	20.2	1	< 0.001		
Ex-drinker	13,239	3.6	6783	3.6	18.9	0.95 (0.92–0.97)			
Current drinker									
light	143,351	39.1	72,291	39.2	18.4	0.93 (0.90–0.95)			
moderate	16,097	4.4	8198	4.3	18.9	0.95 (0.91–0.99)			
heavy	19,720	5.4	10,777	4.7	22.8	1.09 (1.07–1.12)			
amount unknown	10,869	3.0	5772	2.8	20.3	1.01 (0.96–1.05)			
Missing	86,858	23.7							
Medical history									
Diabetes	70,990	19.4	34,520	19.9	17.3	0.87 (0.85–0.88)	< 0.001	0.83 (0.82–0.84)	< 0.001
Chronic kidney disease	25,278	6.9	12,243	5.7	21.4	1.01 (0.98–1.05)	< 0.001		
Chronic liver disease	6440	1.8	3395	1.4	23.5	1.12 (1.08–1.16)	< 0.001		
IMD quintile									
1 (least deprived)	40,769	11.1	21,052	11.7	18.0	1	< 0.001		
2	43,256	11.8	22,097	11.9	18.6	1.01 (0.97–1.06)			
3	48,504	13.2	24,908	13.1	19.1	1.03 (0.98–1.09)			
4	187,806	51.2	98,030	46.1	21.3	1.11 (1.06–1.17)			
5 (most deprived)	46,436	12.7	23,719	12.6	18.8	1.03 (0.97–1.09)			
Calendar year									
2005–07	155,758	42.5	86,412	56.6	15.3	1	< 0.001		
2008–10	124,788	34.0	67,538	30.1	22.5	1.27 (1.26–1.29)			
2011–13	86,225	23.5	35,856	8.7	41.3	1.52 (1.50–1.55)			

^a^Events = first cessation of statin therapy defined as a no further prescriptions 90 days after the expected end date of the last prescription

^b^Cox model with time since study entry (i.e. statin initiation) as the timescale

^c^Adjusted for a priori confounders [age (time-updated), gender, body mass index, smoking status and diabetes (time-updated)]. No other covariates changed the association between cancer survivorship and statin cessation by more than 10%.

All regression models are restricted to patients with complete data for a-priori confounders

**Fig. 3:**
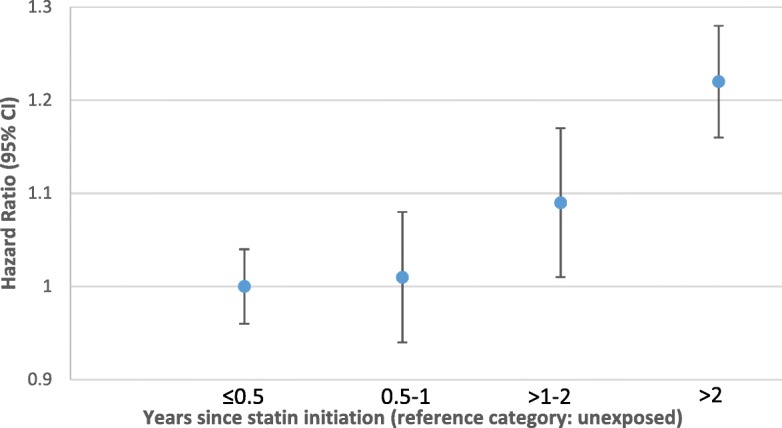
Association between cancer survivorship^i^ and statin discontinuation, by time since statin initiation (index date timescale) ^ii^. Legend: [i] cancer survivors (> 1 year after a cancer diagnosis) compared to never cancer controls. [ii] From a Cox proportional hazards model with time since statin initiation as the timescale, adjusted for ‘current’ age, gender, BMI, smoking and diabetes mellitus (time updated)

Only the a priori confounders (age, sex, BMI, smoking, diabetes status) were included in the final model as none of the other covariates changed the HR for cancer survivorship by more than 10%. The maximum change was 3.6% with the inclusion of calendar year (HR = 1.02, 0.98–1.06). The final adjusted Cox regression model provided evidence that the risk of statin discontinuation was higher in cancer survivors than controls (HR 1.07 95% CI: 1.01–1.12; *p* = 0.02). The same model built with age as the underlying timescale and adjusted for time since statin initiation gave similar results (HR = 1.07, 1.01–1.13, p = 0.02). There was no evidence of interaction between cancer survivorship and calendar period (p-interaction = 0.44) or of differences in the association according to time since cancer diagnosis (*p* = 0.70). However, the association between cancer survivorship status and statin discontinuation appeared to change with time since statin initiation (i.e. evidence of non-proportional hazards, *p* < 0.001): there was no evidence of an effect of cancer survivorship on statin discontinuation in the first 6 months (HR = 1.00 (0.96–1.04)) or at 6 months to 1 year of therapy (HR = 1.01 (0.94–1.08)); from the second year of therapy onwards, there was good evidence that the risk of statin discontinuation rate was higher among cancer survivors (HR = 1.09 95% CI = 1.02–1.17; *p* = 0.008 for the 2nd year of therapy, and HR = 1.22 95% CI =1.16–1.28; *p* < 0.001 for > 2 years after initiation, Fig. 3).

## Discussion

There was a low uptake of statins among cancer survivors with a high recorded cardiovascular risk, with only 23% starting a statin within 31 days of their high risk score record; however, there was no evidence of any difference in uptake between cancer survivors and non-cancer controls, after adjusting for age and sex, either overall or within specific calendar periods. Cancer survivors did appear to be more likely to discontinue statins than controls, in particular after the first year since statin initiation, with the risk of discontinuation 9% (95% CI 2–17%) higher during the second year since statin initiation (*p* = 0.013) and 22% (95% CI 16–28%) higher after 2 years or more on therapy (p < 0.001).

This is the first large observational database study to report on initiation of statin therapy in cancer survivors. Neither are there any directly comparable studies on statin cessation rates in cancer survivors versus non-cancer or general population controls. However, our finding of a greater tendency for cancer survivors to stop statin therapy is in line with a self controlled retrospective cohort study in 36,149 breast cancer patients that used a large health care claims database to compare adherence to oral medications in the 1 year prior to diagnosis with 0.5–1.5 years after diagnosis [25]. The study found adherence to hyperlipidemia medications (based on medication possession ratio) declined from 83.2 to 57.1% (*P* < 0.001). Adherence to long term medications including statins tends to wane with time [26, 27], which might explain the findings from Yang et al.’s after vs before comparison. Statin cessation rates for controls in this study were higher than an earlier study in CPRD (20.1 vs. 13 per 100 person years, respectively), possibly explained by the earlier study excluding people with < 2 statin prescriptions and those with < 1 year of follow-up after their first statin script [28]. One other retrospective cohort study compared statin discontinuation by indication (primary versus secondary prevention) in 539 poor prognosis cancer patients [29]. Over 60% of patients continued filling statin scripts during the 2 years after diagnosis and use was similar regardless of indication. The study can only be generalised to poor prognosis patients and those who start statin therapy before a cancer diagnosis, was underpowered to compare number of refills after diagnosis, and lacked a control group.

Our cohort study was large in size, with sufficient power to produce precise estimates and detect small associations. CPRD is broadly representative of the UK population in terms of age, sex and ethnicity [20]. Cancer diagnoses in CPRD have been previously validated by comparing with national cancer registration and hospitalization and death certificate data: > 90% of cancers in CPRD were confirmed in other data sources, and > 90% of nationally registered cancers were captured in CPRD [30, 31]. Statin prescribing is likely to be extremely well captured in CPRD because the vast majority of statin prescribing takes place in primary care, and GP prescription data are captured automatically at the point of issue.

Our study also has some limitations. There were some missing data in BMI and smoking, leading to omission of patients from the statin cessation regression analysis (in which only complete cases were included); this could lead to selection bias if the association between the exposure, outcome and covariates was different in patients with and without missing data. However, the amount of missing data for BMI and smoking was low (≤ 3.1%) and this is unlikely to have significantly impacted our results.

Despite the excellent prescribing data in CPRD, statin initiation might have been underestimated because over the counter therapy (OTC) is not captured in CPRD. However based on OTC sales and consumer interviews, this is not expected to be large issue [32]. Equally, in the statin initiation analysis, it was impossible to decipher from the data available whether a statin was offered and refused by the patient versus not offered. Misclassification of statin cessation may also have occurred for individuals who did not fill the statin prescription and individuals who received the drug but not take it. This may lead to an underestimation of statin cessation in cancer survivors and controls. To our knowledge, there is no evidence that this would differ between groups, so this non-differential misclassification may have led to an underestimation of the difference in cessation between the groups. While the analyses of statin initiation and cessation attempted to control for confounding, some potential confounding factors were not consistently available for individual patients, including ethnicity, and family history of CVD, so there is some potential for residual confounding. Ethnic minorities tend to have lower cancer rates [33] and are more likely to discontinue statins [34] which could also produce negative confounding, not controlled for in this study.

During cancer treatment and survivorship, patients are likely to visit their GP more regularly and measurement of individual cardiovascular risk factors is increased. This represents an opportunity to re-inforce the importance of preventative measures in a patient group that is thought to be at increased risk of CVD. Our findings suggest that this opportunity has been missed over the study period (2005–2013). Initiatives such as cancer treatment summaries and survivorship care plans aim to increase GP and patient awareness of potential adverse events associated with cancer treatment [35, 36]. Better implementation of these initiatives may improve uptake of measures designed to prevent CVD.

Our study highlights opportunities for further research. We considered cancer survivors in a single group, but future research might look at whether our results are dependent upon type of cancer history. Our descriptive analyses demonstrated suboptimal levels of cardiovascular risk score recording in both cancer survivors and never cancer controls. Future studies are warranted estimating the number of cancer survivors who are not prescribed statins although they are at high risk of CVD, whether or not they have a risk score recorded. Qualitative studies might be carried out to further investigate the reasons for similarly low levels of statin initiation and slightly higher statin cessation rates among cancer survivors after 1 year compared to the general population. Other aspects of cardiovascular risk prevention should also be assessed, including initiation of antihypertensive therapies in at risk patients.

## Conclusions

We observed low uptake of statins in both cancer survivors and controls with no history of cancer, with no evidence of difference between the two groups. Cessation of statin use is substantial and may be somewhat higher after 1 year of treatment in cancer survivors than in the general population. Further research might explore the reasons for this and investigate a wider range of preventative measures. There may be an opportunity to mitigate the suspected higher cardiovascular risk in the growing population of cancer survivors by improving uptake of lipid-lowering treatment and persistence on therapy.

## Additional files

Additional file 1: **Table S1.** Guidelines for the primary prevention of cardiovascular disease using statins and antihypertensive medicines (1998–2014). (DOCX 20 kb)

Additional file 2: **Table S2.** Code list for the identification of a CVD event recorded in the CPRD database. (DOCX 52 kb)

Additional file 3: **Table S3.** Code list for the identification of a cardiovascular risk event recorded in the CPRD database. (DOCX 20 kb)

Additional file 4: **Table S4.** Product codes for the identification of statin therapy. (DOCX 26 kb)

Additional file 5: **Table S5.** Proportion (%) of individuals with a blood pressure, cholesterol or cardiovascular risk score recorded in the past 5 years, by age and gender. (DOCX 20 kb)

Additional file 6: ISAC protocol 16_163. (PDF 735 kb)

## Abbreviations

BMI: Body mass index
CKD: Chronic kidney disease
CPRD: Clinical Practice Research Datalink
CVD: Cardiovascular disease
GP: General practitioner
HDL: High-density lipoprotein
HR: Hazard ratio
NHS: National Health Service
OTC: Over the counter therapy
UK: United Kingdom

## References

[1] Allemani C, Weir HK, Carreira H, Harewood R, Spika D, Wang X-S (2015). Global surveillance of cancer survival 1995-2009: analysis of individual data for 25,676,887 patients from 279 population-based registries in 67 countries (CONCORD-2). Lancet.

[2] Quaresma M, Coleman MP, Rachet B (2015). 40-year trends in an index of survival for all cancers combined and survival adjusted for age and sex for each cancer in England and Wales, 1971-2011: a population-based study. Lancet.

[3] Daher IN, Daigle TR, Bhatia N, Durand J-B (2012). The prevention of cardiovascular disease in cancer survivors. Texas Hear Inst J.

[4] Macmillan Cancer Support. Throwing light on the consequences of cancer and its treatment (report) [Internet]. 2013 [cited 2018 Jan 9]. Available from: https://www.macmillan.org.uk/documents/aboutus/research/researchandevaluationreports/throwinglightontheconsequencesofcanceranditstreatment.pdf.

[5] Carver JR, Shapiro CL, Ng A, Jacobs L, Schwartz C, Virgo KS (2007). American Society of Clinical Oncology clinical evidence review on the ongoing care of adult cancer survivors: cardiac and pulmonary late effects. J Clin Oncol. American Society of Clinical Oncology.

[6] Department of Health. Living with and beyond cancer: taking action to improve outcomes (an update to the 2010 The National Cancer Survivorship Initiative Vision) [Internet]. Dep. Heal. 2013 [cited 2017 Dec 12]. Available from: https://www.gov.uk/government/uploads/system/uploads/attachment_data/file/181054/9333-TSO-2900664-NCSI_Report_FINAL.pdf.

[7] Armenian SH, Xu L, Ky B, Sun C, Farol LT, Pal SK (2016). Cardiovascular disease among survivors of adult-onset Cancer: a community-based retrospective cohort study. J Clin Oncol.

[8] Patnaik JL, Byers T, DiGuiseppi C, Dabelea D, Denberg TD (2011). Cardiovascular disease competes with breast cancer as the leading cause of death for older females diagnosed with breast cancer: a retrospective cohort study. Breast Cancer Res BioMed Central.

[9] Hanrahan EO, Gonzalez-Angulo AM, Giordano SH, Rouzier R, Broglio KR, Hortobagyi GN (2007). Overall survival and cause-specific mortality of patients with stage T1a,bN0M0 breast carcinoma. J Clin Oncol.

[10] Chang H-M, Okwuosa TM, Scarabelli T, Moudgil R, Yeh ETH (2017). Cardiovascular complications of Cancer therapy: best practices in diagnosis, prevention, and management: part 2. J Am Coll Cardiol.

[11] Chang H-M, Moudgil R, Scarabelli T, Okwuosa TM, Yeh ETH (2017). Cardiovascular complications of Cancer therapy: best practices in diagnosis, prevention, and management: part 1. J Am Coll Cardiol.

[12] Nathan PC, Amir E, Abdel-Qadir H (2016). Cardiac outcomes in survivors of pediatric and adult cancers. Can J Cardiol Elsevier.

[13] Weaver KE, Foraker RE, Alfano CM, Rowland JH, Arora NK, Bellizzi KM (2013). Cardiovascular risk factors among long-term survivors of breast, prostate, colorectal, and gynecologic cancers: a gap in survivorship care?. J Cancer Surviv.

[14] World Health Organisation. Prevention of Cardiovascular Disease: guidelines for assessment and management of total cardiovascular risk [Internet]. Geneva; 2007 [cited 2018 Jan 12]. Available from: http://apps.who.int/iris/bitstream/10665/43685/1/9789241547178_eng.pdf.

[15] Joint British Societies (2005). JBS 2: joint British societies’ guidelines on prevention of cardiovascular disease in clinical practice. Heart.

[16] Robson J, Dostal I, Sheikh A, Eldridge S, Madurasinghe V, Griffiths C (2016). The NHS health check in England: an evaluation of the first 4 years. BMJ Open.

[17] Forster AS, Dodhia H, Booth H, Dregan A, Fuller F, Miller J (2015). Estimating the yield of NHS health checks in England: a population-based cohort study. J Public Health (Oxf).

[18] Chang KC-M, Soljak M, Lee JT, Woringer M, Johnston D, Khunti K (2015). Coverage of a national cardiovascular risk assessment and management programme (NHS health check): retrospective database study. Prev Med (Baltim).

[19] van Staa T-P, Smeeth L, Ng ES-W, Goldacre B, Gulliford M (2013). The efficiency of cardiovascular risk assessment: do the right patients get statin treatment?. Heart.

[20] Herrett E, Gallagher AM, Bhaskaran K, Forbes H, Mathur R, van Staa T (2015). Data resource profile: clinical practice research datalink (CPRD). Int J Epidemiol.

[21] NHS Digital. Patients Registered at a GP Practice, December 2017 [Internet]. 2017 [cited 2018 Jan 12]. Available from: https://digital.nhs.uk/catalogue/PUB30171.

[22] Bhaskaran K, Douglas I, Forbes H, dos-Santos-Silva I, Leon DA, Smeeth L (2014). Body-mass index and risk of 22 specific cancers: a population-based cohort study of 5·24 million UK adults. Lancet Elsevier.

[23] UK Data Service Census Support. Deprivation data [Internet]. [cited 2018 Jan 10]. Available from: https://census.ukdataservice.ac.uk/get-data/related/deprivation.

[24] Trusler D. (2011). Statin prescriptions in UK now total a million each week. BMJ.

[25] Yang J, Neugut AI, Wright JD, Accordino M, Hershman DL (2016). Nonadherence to Oral medications for chronic conditions in breast Cancer survivors. J Oncol Pract.

[26] Maningat P, Gordon BR, Breslow JL (2013). How do we improve patient compliance and adherence to long-term statin therapy?. Curr Atheroscler Rep.

[27] Elliott RA, Boyd MJ, Salema N-E, Davies J, Barber N, Mehta RL (2016). Supporting adherence for people starting a new medication for a long-term condition through community pharmacies: a pragmatic randomised controlled trial of the new medicine service. BMJ Qual Saf.

[28] Vinogradova Y, Coupland C, Brindle P, Hippisley-Cox J (2016). Discontinuation and restarting in patients on statin treatment: prospective open cohort study using a primary care database. BMJ.

[29] Bayliss EA, Bronsert MR, Reifler LM, Ellis JL, Steiner JF, McQuillen DB (2013). Statin prescribing patterns in a cohort of cancer patients with poor prognosis. J Palliat Med.

[30] Boggon R, van Staa TP, Chapman M, Gallagher AM, Hammad TA, Richards MA (2013). Cancer recording and mortality in the general practice research database and linked cancer registries. Pharmacoepidemiol Drug Saf.

[31] Dregan A, Moller H, Murray-Thomas T, Gulliford MC (2012). Validity of cancer diagnosis in a primary care database compared with linked cancer registrations in England. Population-based cohort study. Cancer Epidemiol Elsevier Ltd.

[32] Will CM, Weiner K (2015). The drugs don’t sell: DIY heart health and the over-the-counter statin experience. Soc Sci Med.

[33] Lowth M. Diseases and Different Ethnic Groups [Internet]. Patient Prof. Ref. 2015 [cited 2018 Jan 10]. Available from: https://patient.info/doctor/diseases-and-different-ethnic-groups.

[34] Mann DM, Woodward M, Muntner P, Falzon L, Kronish I (2010). Predictors of nonadherence to statins: a systematic review and meta-analysis. Ann Pharmacother NIH Public Access.

[35] Jefford M, Rowland J, Grunfeld E, Richards M, Maher J, Glaser A (2013). Implementing improved post-treatment care for cancer survivors in England, with reflections from Australia, Canada and the USA. Br J Cancer.

[36] Walter FM, Usher-Smith JA, Yadlapalli S, Watson E (2015). Caring for people living with, and beyond, cancer: an online survey of GPs in England. Br J Gen Pract.

