# Supplementary material for: Statin use in cancer survivors versus the general population: cohort study using primary care data from the UK clinical practice research datalink
Source: BMC Cancer. 2018 Oct 22;18:1018. doi: 10.1186/s12885-018-4947-8 (PMC6196462; doi:10.1186/s12885-018-4947-8)
Supplement: Supplementary file 1 — Table S1. Guidelines for the primary prevention of cardiovascular disease using statins and antihypertensive medicines (1998–2014). (DOCX 20 kb) [file 12885_2018_4947_MOESM1_ESM.docx]

**Table S1: Guidelines for the primary prevention of cardiovascular disease using statins and antihypertensive medicines (1998-2014)**

| Year published | Guideline | Indication for statin/AHT based on high CVD risk score | Indication for statin/AHT based on high risk (no CVD risk assessment required) |
| --- | --- | --- | --- |
| 1998 | JBS1: Joint British Societies’ guidelines on prevention of cardiovascular disease in clinical practice | As a minimum all individuals with an absolute CHD risk of 30% or more over 10 years.  When it has been shown that those at highest risk have been effectively targeted the scientific evidence justifies a progressive expansion of coronary prevention from 30% down to 15% absolute CHD risk, linked to NHS resources needed to deliver effective preventive care. | Indication for AHT  severe hypertension (systolic > 160 mm Hg and/or diastolic > 100 mm Hg)  Indication for statin  familial hypercholesterolaemia or other inherited dyslipidaemia  diabetes mellitus with associated target organ damage. |
| 2000 | National Service Framework. Chapter 2. Preventing coronary heart disease in high risk patients. | CHD risk greater than 30% over ten years |  |
| 2004 | NICE guidelines [CG18]. Essential hypertension: managing adult patients in primary care | Indication for AHT:  Persistent blood pressure ≥140/90mmHg AND 10-year cardiovascular risk score ≥20% OR 10-year CHD score ≥15% | Indication for AHT:  Persistent high BP of ≥160/100mmHg  Persistent blood pressure ≥140/90mmHg AND existing CVD or target organ damage |
| 2005 | JBS 2: Joint British Societies' guidelines on prevention of cardiovascular disease in clinical practice. | Indication for AHT:  All those with CVD risk ≥20% over 10 years (i.e. high risk) AND  Stage 1 hypertension (BP ≥140/90mmHg)  Indication for statin:  All those with CVD risk ≥20% over 10 years (i.e. high risk). | Indication for AHT:  elevated blood pressure > 160 mm Hg systolic or > 100 mm Hg diastolic, or lesser degrees of blood pressure elevation with target organ damage  Indication for statin:  elevated total cholesterol to high density lipoprotein (HDL) cholesterol ratio > 6.0  familial dyslipidaemia, such as familial hypercholesterolaemia or familial combined hyperlipidaemia.  >40yo and type 1 or 2 DM |
| 2005 | World Health Organisation (WHO). Prevention of cardiovascular disease: guidelines for assessment and management of total cardiovascular risk. | 10-year total CVD risk thresholds for intensive intervention:  high-resource setting: 20%  medium-resource setting: 30%  low-resource setting: 40% | total cholesterol ≥ 8 mmol/l (320 mg/dl) or low-density lipoprotein (LDL) cholesterol ≥ 6 mmol/l (240 mg/dl) or TC/HDL-C ratio > 8;  persistent raised blood pressure (> 160–170/100–105 mmHg) (38–41, 43, 83);  type 1 or 2 diabetes, with overt nephropathy or other significant renal disease;  patients with known renal failure or renal impairment. |
| 2006 | NICE guidelines [CG34] Hypertension: management of hypertension in adults in primary care. | Indication for AHT:  Persistent blood pressure ≥140/90mmHg AND 10-year cardiovascular risk score ≥20% | Indication for AHT:  Persistent high BP of ≥160/100mmHg (any age)  Persistent blood pressure ≥140/90mmHg AND existing CVD or target organ damage |
| 2008 | NICE guidelines [CG67] Lipid modification: Cardiovascular risk assessment and the modification of blood lipids for the primary and secondary prevention of cardiovascular disease | Indication for statin:  Framingham score >20% | Indication for statin:  Age 75+  Familial hypercholesterolaemia  Use clinical assessment for people for whom an appropriate risk calculator is not available or appropriate^[[1]](#footnote-1)^ |
| 2011 | NICE guidelines [CG127] Hypertension in adults: diagnosis and management | Indication for AHT:  Stage 1 hypertension (≥140/90mmHg) AND 10-year cardiovascular risk score ≥20% | Indication for AHT:  Stage 2 hypertension ≥160/100mmHg (any age)  <80 years AND Stage 1 hypertension AND 1 or more of the following:  Target organ damage (heart/kidneys)  Established CVD  Renal disease  diabetes |
| 2014 | NICE guidelines [CG181] Cardiovascular disease: risk assessment and reduction, including lipid modification | Indication for statin^i^:  QRisk2 score >10% | Indication for statin:  Age 85+  Type I diabetes and >40 years (regardless of CVD risk score)  CKD (estimated glomerular filtration rate (eGFR) less than 60 ml/min/1.73m2 and/or albuminuria.  Familial hypercholesterolaemia |

1. Recognise that standard CVD risk scores will underestimate risk in people who have additional risk because of underlying medical conditions or treatments. These groups include:

   - people treated for HIV
   - people with serious mental health problems
   - people taking medicines that can cause dyslipidaemia such as antipsychotic medication, corticosteroids or immunosuppressant drugs
   - people with autoimmune disorders such as systemic lupus erythematosus, and other systemic inflammatory disorders.

   [↑](#footnote-ref-1)
