# Supplementary material for: Statin use in cancer survivors versus the general population: cohort study using primary care data from the UK clinical practice research datalink
Source: BMC Cancer. 2018 Oct 22;18:1018. doi: 10.1186/s12885-018-4947-8 (PMC6196462; doi:10.1186/s12885-018-4947-8)
Supplement: Supplementary file 2 — Table S2. Code list for the identification of a CVD event recorded in the CPRD database. (DOCX 52 kb) [file 12885_2018_4947_MOESM2_ESM.docx]

**Table S2: Code list for the identification of a CVD event recorded in the CPRD database**

| medcode | readterm | medcode | readterm |
| --- | --- | --- | --- |
| 6305 | H/O: CVA | 47562 | Open embolectomy of common iliac artery |
| 54535 | Stenocardia | 31941 | Rupture of syphilitic cerebral aneurysm |
| 5871 | H/O: stroke | 39780 | Other open operations on carotid artery |
| 1204 | Heart attack | 28651 | Iliac and femoral artery operations NOS |
| 6853 | Claudication | 73822 | Reconstruction of common femoral artery |
| 9561 | Ischaemic toe | 44097 | Transluminal operations on iliac artery |
| 12804 | Stable angina | 62107 | Type II diabetes mellitus with gangrene |
| 13185 | Angina control | 23361 | Late effects of cerebrovascular disease |
| 7780 | Left sided CVA | 27661 | Extradural haemorrhage following injury |
| 13566 | Attack - heart | 64798 | Other bypass of bifurcation of aorta NOS |
| 19655 | Angina at rest | 11766 | Other emergency bypass of femoral artery |
| 98174 | Ischaemic foot | 41768 | Ilio-femoral prosthetic cross over graft |
| 17307 | Angina at rest | 57822 | Other open operation on iliac artery NOS |
| 1430 | Angina pectoris | 55402 | Open embolectomy of bifurcation of aorta |
| 1431 | Unstable angina | 52462 | Transluminal procedure on femoral artery |
| 12833 | Right sided CVA | 51124 | Ligation of aneurysm of popliteal artery |
| 1298 | CVA unspecified | 20892 | Other open operations on popliteal artery |
| 34135 | H/O: CVA/stroke | 25910 | Transluminal operations on carotid artery |
| 7347 | Unstable angina | 6155 | Stroke due to cerebral arterial occlusion |
| 1735 | Aortic aneurysm | 69124 | IDDM with peripheral circulatory disorder |
| 18125 | Nocturnal angina | 52008 | Other open operations on vertebral artery |
| 4656 | Crescendo angina | 15007 | Replacement of carotid artery using graft |
| 18118 | Worsening angina | 39749 | Operation on aneurysm of iliac artery NEC |
| 20095 | Angina decubitus | 44430 | Endarterectomy of common iliac artery NEC |
| 34758 | Cerebral embolus | 43292 | Arteriosclerotic dementia with depression |
| 7696 | Syncope anginosa | 46545 | Cerebral haemorrhage following injury NOS |
| 66388 | Status anginosus | 52473 | Transluminal procedure on popliteal artery |
| 26863 | New onset angina | 32854 | Acute posterolateral myocardial infarction |
| 20510 | [SO]Carotid body | 55877 | Transluminal operation on iliac artery NOS |
| 17133 | Mural thrombosis | 6116 | CVA - Cerebrovascular accident unspecified |
| 1414 | Angina on effort | 40732 | Other bypass of superficial femoral artery |
| 34328 | Refractory angina | 59534 | H/O: Peripheral vascular disease procedure |
| 1826 | Ischaemia of legs | 11430 | Thoracoabdominal aortic aneurysm, ruptured |
| 10792 | Stroke monitoring | 95503 | Replacement of aneurysmal iliac artery NOS |
| 15019 | Cerebral embolism | 31822 | Other emergency bypass of segment of aorta |
| 26966 | ECG: S-T elevation | 70235 | Other open operation on carotid artery NOS |
| 8659 | [SO]Carotid artery | 54071 | Revision of reconstruction involving aorta |
| 8692 | Cerebellar disease | 30989 | Femoro-femoral prosthetic cross over graft |
| 8568 | Cardiac syndrome X | 22016 | Femoro-femoral prosthetic cross over graft |
| 6253 | Stroke unspecified | 39776 | Other emergency bypass of popliteal artery |
| 15349 | Angina control NOS | 56803 | NIDDM with peripheral circulatory disorder |
| 94504 | Door to needle time | 38907 | Other specified peripheral vascular disease |
| 45960 | Antianginal therapy | 91775 | Intracranial bypass from carotid artery NEC |
| 9555 | Post infarct angina | 30202 | Intracerebral haemorrhage, intraventricular |
| 2491 | Coronary thrombosis | 52968 | Other cerebral haemorrhage following injury |
| 28554 | Angina pectoris NOS | 28840 | Operation on aneurysm of femoral artery NEC |
| 6308 | Ischaemic leg ulcer | 52357 | Other specified other bypass of iliac artery |
| 25842 | Angina pectoris NOS | 17767 | Abdominal aortic aneurysm which has ruptured |
| 16517 | Cerebral thrombosis | 41703 | Transluminal operation on carotid artery NOS |
| 52246 | Stroke group member | 53580 | Other open operations on common iliac artery |
| 7912 | Pontine haemorrhage | 96809 | Reconstruction of superficial femoral artery |
| 3778 | Aorto biiliac graft | 55602 | Occlusion and stenosis of cerebellar arteries |
| 12986 | Prinzmetal's angina | 63238 | Other emergency bypass of deep femoral artery |
| 47642 | Wallenberg syndrome | 57315 | Intracerebral haemorrhage, multiple localized |
| 6872 | Aortic aneurysm NOS | 31805 | Other and unspecified intracranial haemorrhage |
| 15661 | Dressler's syndrome | 52358 | Replacement of aneurysmal bifurcation of aorta |
| 25615 | Brainstem infarction | 60212 | Other open operations on common femoral artery |
| 39655 | Impending infarction | 10962 | Exception reporting: stroke quality indicators |
| 30330 | Acute Q-wave infarct | 69346 | Other replacement of aneurysmal femoral artery |
| 51504 | Epidural haemorrhage | 16034 | Aortic aneurysm without mention of rupture NOS |
| 16993 | H/O: aortic aneurysm | 59187 | Transluminal operations on common iliac artery |
| 32450 | Ischaemic chest pain | 65286 | Revision of reconstruction of popliteal artery |
| 29902 | Angina decubitus NOS | 5387 | Other specified anterior myocardial infarction |
| 46227 | ECG: Q wave abnormal | 50894 | Other specified reconstruction of iliac artery |
| 31595 | Cortical haemorrhage | 15302 | Peripheral arterial embolism and thrombosis NOS |
| 6827 | Peripheral ischaemia | 1469 | Stroke and cerebrovascular accident unspecified |
| 32959 | Seen in stroke clinic | 72562 | Subsequent myocardial infarction of other sites |
| 13571 | Thrombosis - coronary | 42283 | Other cerebral haemorrhage following injury NOS |
| 5602 | Cerebellar infarction | 62818 | Endarterectomy and patch repair of iliac artery |
| 569 | Infarction - cerebral | 4325 | Other specified peripheral vascular disease NOS |
| 36854 | Coronary artery spasm | 90549 | Endovascular stenting of aortic bifurcation NEC |
| 101866 | Vascular claudication | 73961 | [X]Other specified peripheral vascular diseases |
| 19542 | Angina control - good | 51759 | Occlusion and stenosis of middle cerebral artery |
| 15373 | Angina control - poor | 68748 | Postoperative myocardial infarction, unspecified |
| 17347 | Leg artery operations | 43108 | Other replacement of aneurysmal segment of aorta |
| 14895 | Aorto bifemoral graft | 47607 | CVA - cerebrovascular accident in the puerperium |
| 36523 | Preinfarction syndrome | 68329 | Other specified reconstruction of carotid artery |
| 32556 | Diabetes with gangrene | 44439 | Other replacement of aneurysmal popliteal artery |
| 13564 | Cerebellar haemorrhage | 52869 | Reconstruction of femoral artery with vein graft |
| 1736 | Aortic aneurysm repair | 92925 | Y graft of abdominal Aortic aneurysm (emergency) |
| 22677 | Carotid artery disease | 12229 | Acute ST segment elevation myocardial infarction |
| 52241 | [SO]Carotid artery NEC | 63408 | Tube graft abdominal Aortic aneurysm (emergency) |
| 1318 | Aortic atherosclerosis | 95976 | Endovascular stenting of aorto-uniiliac aneurysm |
| 11048 | Variant angina pectoris | 67083 | Reconstruction of femoral or popliteal artery OS |
| 23942 | Basilar artery syndrome | 97109 | Endovascular stenting for aorto-uniiliac aneurysm |
| 3149 | Cerebral infarction NOS | 28777 | Endarterectomy and patch repair of femoral artery |
| 2418 | Cerebrovascular disease | 60499 | Insulin dependent diabetes mellitus with gangrene |
| 2652 | Carotid artery stenosis | 40053 | Generalised ischaemic cerebrovascular disease NOS |
| 41221 | Acute septal infarction | 62301 | Replacement of aneurysmal bifurcation of aorta OS |
| 28736 | Acute atrial infarction | 45809 | Subsequent myocardial infarction of anterior wall |
| 52705 | ECG: lateral infarction | 6617 | Reconstruction of femoral or popliteal artery NOS |
| 98145 | ABCD2 stroke risk score | 28314 | Left sided intracerebral haemorrhage, unspecified |
| 56279 | Stroke in the puerperium | 35916 | Endarterectomy and patch repair of carotid artery |
| 2156 | Stenosis, carotid artery | 49273 | Revision of reconstruction involving iliac artery |
| 18912 | Subdural haemorrhage NOS | 38609 | Subsequent myocardial infarction of inferior wall |
| 18686 | Stroke/CVA annual review | 12555 | Generalised ischaemic cerebrovascular disease NOS |
| 66873 | H/O: Stroke in last year | 41835 | Postoperative subendocardial myocardial infarction |
| 4240 | Carotid artery occlusion | 57527 | Occlusion and stenosis of anterior cerebral artery |
| 68357 | Microinfarction of heart | 69519 | Reconstruction of popliteal artery with vein graft |
| 17336 | Open femoral embolectomy | 17960 | Carotid, cerebral and subclavian artery operations |
| 57495 | Infarction - precerebral | 19201 | Right sided intracerebral haemorrhage, unspecified |
| 62270 | ECG: Q wave pathological | 24692 | Other bypass of femoral artery or popliteal artery |
| 5221 | Cardiac enzymes abnormal | 66761 | Replacement of aneurysmal bifurcation of aorta NOS |
| 32447 | Basilar artery occlusion | 93468 | Type 1 diabetes mellitus with peripheral angiopathy |
| 15252 | Brainstem infarction NOS | 49319 | Other open operations on superficial femoral artery |
| 19477 | Arteriosclerotic dementia | 97030 | Endovascular stenting of suprarenal aortic aneurysm |
| 46316 | Basal nucleus haemorrhage | 52695 | Endarterectomy and patch repair of popliteal artery |
| 36579 | [SO]Common carotid artery | 65770 | Occlusion and stenosis of posterior cerebral artery |
| 91515 | [SO]Common carotid artery | 18038 | Revision of reconstruction involving femoral artery |
| 1517 | Intermittent claudication | 50594 | Multiple and bilateral precerebral artery syndromes |
| 42465 | Dacron aortoiliac Y graft | 60699 | Type 2 diabetes mellitus with peripheral angiopathy |
| 70440 | Pain to thrombolysis time | 37806 | Type 2 diabetes mellitus with peripheral angiopathy |
| 14797 | Extremity artery atheroma | 54212 | Non-insulin-dependent d m with peripheral angiopath |
| 97001 | Cardiac troponin positive | 63605 | Profundoplasty and patch repair of popliteal artery |
| 12634 | Carotid artery dissection | 55445 | Other replacement of aneurysmal segment of aorta OS |
| 33377 | Vertebral artery syndrome | 19412 | Subarachnoid haemorrhage from middle cerebral artery |
| 5051 | Intracerebral haemorrhage | 55324 | Other specified other open operation on iliac artery |
| 54251 | Preinfarction syndrome NOS | 31613 | Emergency replacement of aneurysmal segment of aorta |
| 7783 | ECG: myocardial infarction | 43648 | Emergency femoro-femoral prosthetic cross over graft |
| 14782 | Angina control - improving | 31338 | Reconstruction of femoral artery or popliteal artery |
| 42248 | Discharge from stroke serv | 30421 | Cardiac rupture following myocardial infarction (MI) |
| 17322 | Cerebellar stroke syndrome | 51720 | Transluminal procedure on superficial femoral artery |
| 93459 | [X]Other lacunar syndromes | 1867 | Abdominal aortic aneurysm without mention of rupture |
| 24385 | Chronic cerebral ischaemia | 31060 | Intracerebral haemorrhage in hemisphere, unspecified |
| 4152 | Thrombosis, carotid artery | 10562 | Acute non-ST segment elevation myocardial infarction |
| 40847 | Vertebral artery occlusion | 54899 | Type II diabetes mellitus with peripheral angiopathy |
| 8443 | Brain stem stroke syndrome | 55825 | Other specified other bypass of bifurcation of aorta |
| 45521 | Juxtarenal aortic aneurysm | 46125 | Emergency replacement of aneurysmal popliteal artery |
| 5185 | Lateral medullary syndrome | 97606 | Other emergency bypass of superficial femoral artery |
| 61256 | Repair of iliac artery NEC | 36651 | Other replacement of aneurysmal segment of aorta NOS |
| 29300 | Angina control - worsening | 92036 | [X]Occlusion and stenosis of other cerebral arteries |
| 59032 | ECG: myocardial infarct NOS | 46166 | Subsequent myocardial infarction of unspecified site |
| 55401 | ECG: subendocardial infarct | 51138 | Sequelae/other + unspecified cerebrovascular diseases |
| 10062 | Cerebrovascular disease NOS | 98642 | Multiple and bilateral precerebral arterial occlusion |
| 33499 | Pure motor lacunar syndrome | 42640 | Other bypass of femoral artery or popliteal artery OS |
| 241 | Acute myocardial infarction | 7138 | [V]Personal history of cerebrovascular accident (CVA) |
| 73901 | [X]Cerebrovascular diseases | 40401 | Non-insulin dependent diabetes mellitus with gangrene |
| 96117 | [SO]External carotid artery | 10827 | Percutaneous transluminal angioplasty of iliac artery |
| 28166 | Dacron aortofemoral Y graft | 66930 | Percutaneous transluminal embolectomy of iliac artery |
| 60102 | [SO]Internal carotid artery | 24677 | Other open operation on femoral or popliteal artery OS |
| 26975 | ECG: antero-septal infarct. | 93959 | Transluminal operations on aneurysmal segment of aorta |
| 8837 | Cerebral arterial occlusion | 63368 | Repair of femoral artery with temporary silastic shunt |
| 26424 | Infarction of basal ganglia | 44023 | Other specified other open operation on carotid artery |
| 9507 | Acute non-Q wave infarction | 46465 | Transluminal operations on femoral or popliteal artery |
| 22447 | [SO]Internal carotid artery | 64446 | Insulin dependent diab mell with peripheral angiopathy |
| 21927 | Other bypass of iliac artery | 28894 | Other specified transluminal operation on iliac artery |
| 20811 | Bypass to carotid artery NEC | 36065 | Endarterectomy and patch repair of common iliac artery |
| 7862 | Traumatic subdural haematoma | 2066 | Other bypass of femoral artery or popliteal artery NOS |
| 41577 | H/O: cerebrovascular disease | 35399 | Diabetes mellitus with peripheral circulatory disorder |
| 19825 | Femoral arteriogram abnormal | 46168 | Other specified operations on iliac and femoral artery |
| 9276 | Acute coronary insufficiency | 29973 | Percutaneous transluminal angioplasty of carotid artery |
| 35119 | Post infarction pericarditis | 90572 | [X]Occlusion and stenosis of other precerebral arteries |
| 15304 | Ruptured aortic aneurysm NOS | 43451 | Sequelae of other nontraumatic intracranial haemorrhage |
| 73022 | Repair of carotid artery NEC | 6256 | Percutaneous transluminal embolectomy of femoral artery |
| 41818 | Carotid A angiogram abnormal | 47538 | Other open operation on femoral or popliteal artery NOS |
| 55870 | Abdominal aortogram abnormal | 40758 | Cereb infarct due unsp occlus/stenos precerebr arteries |
| 30045 | External capsule haemorrhage | 29758 | Acute transmural myocardial infarction of unspecif site |
| 53745 | [X]Other cerebral infarction | 96630 | [X]Intracerebral haemorrhage in hemisphere, unspecified |
| 20284 | Intracranial haemorrhage NOS | 6356 | Percutaneous transluminal angioplasty of femoral artery |
| 33555 | Repair of femoral artery NEC | 45477 | Emergency replacement of aneurysmal segment of aorta OS |
| 40338 | Internal capsule haemorrhage | 65692 | Other emergency bypass of femoral or popliteal artery OS |
| 9565 | [X]Arteriosclerotic dementia | 24126 | Haemopericardium/current comp folow acut myocard infarct |
| 17689 | Silent myocardial infarction | 17326 | Subarachnoid haemorrh from intracranial artery, unspecif |
| 40429 | Acute anteroapical infarction | 66869 | Repair of popliteal artery with temporary silastic shunt |
| 24327 | Ischaemic ulcer diabetic foot | 41583 | Transluminal operation on femoral or popliteal artery OS |
| 42279 | Arteriosclerotic dementia NOS | 71041 | Percutaneous transluminal embolisation of femoral artery |
| 13577 | Other cerebrovascular disease | 27975 | Cerebral infarction due to embolism of cerebral arteries |
| 51767 | Pure sensory lacunar syndrome | 47580 | Percutaneous transluminal insertion stent carotid artery |
| 19348 | [V]Personal history of stroke | 62661 | Other specified transluminal operation on carotid artery |
| 34153 | Axillo-bifemoral bypass graft | 45474 | Emergency replacement of aneurysmal segment of aorta NOS |
| 17872 | Acute anteroseptal infarction | 68320 | Other emergency bypass of femoral or popliteal artery NOS |
| 16260 | Extremity artery atheroma NOS | 39344 | Cereb infarct due cerebral venous thrombosis, nonpyogenic |
| 10794 | Vertebrobasilar insufficiency | 24229 | Percutaneous transluminal insertion of iliac artery stent |
| 3535 | Intracerebral haemorrhage NOS | 52289 | Percutaneous transluminal embolectomy of popliteal artery |
| 12139 | Acute anterolateral infarction | 34375 | Stroke/transient ischaemic attack monitoring third letter |
| 45781 | Precerebral arterial occlusion | 15532 | Bypass bifurcation aorta by anastom aorta to iliac artery |
| 2417 | Vertebro-basilar insufficiency | 6228 | Sequelae of stroke,not specfd as h'morrhage or infarction |
| 18411 | Traumatic extradural haematoma | 68905 | Percutaneous transluminal embolectomy of vertebral artery |
| 34152 | Diabetic peripheral angiopathy | 29112 | Percutaneous transluminal angioplasty of popliteal artery |
| 31876 | Aneurysm of common carotid art | 28753 | Stroke/transient ischaemic attack monitoring first letter |
| 39039 | Reconstruction of iliac artery | 96677 | Traumatic subdural haematoma with open intracranial wound |
| 24540 | Chronic coronary insufficiency | 63711 | Transluminal operation on femoral or popliteal artery NOS |
| 37199 | Carotid artery atherosclerosis | 42115 | Bypass popliteal artery by pop/fem artery anastomosis NEC |
| 53634 | [D]Gangrene of toe in diabetic | 55074 | Percutaneous transluminal angioplasty of vertebral artery |
| 28119 | Repair of popliteal artery NEC | 11074 | Excepted from stroke quality indicators: Informed dissent |
| 28914 | Haemorrhagic stroke monitoring | 33543 | Cerebrl infarctn due/unspcf occlusn or sten/cerebrl artrs |
| 9985 | Left sided cerebral infarction | 34245 | Stroke/transient ischaemic attack monitoring second letter |
| 8181 | Traumatic subdural haemorrhage | 61974 | Other bypass of femoral or popliteal artery by anastomosis |
| 18060 | Other bypass of femoral artery | 66820 | Emerg bypass femoral art by fem/tib art anast c prosth NEC |
| 59602 | Iliac endarterectomy and patch | 2761 | Bypass bifurc aorta by anastom aorta to femoral artery NEC |
| 26972 | ECG:posterior/inferior infarct | 96654 | Emerg repl aneurysm bifurc aorta by anast aorta to fem art |
| 5268 | Insufficiency - basilar artery | 39449 | Coronary thrombosis not resulting in myocardial infarction |
| 28077 | Traumatic cerebral haemorrhage | 43651 | Emerg bypass femoral art by fem/pop art anast c prosth NEC |
| 8935 | Acute inferolateral infarction | 69922 | Emerg repl aneurysm bifurc aorta by anast aorta to iliac a |
| 41825 | Lower limb arteriogram abnorm. | 94482 | [X]Cereb infarct due unsp occlus/stenos precerebr arteries |
| 14658 | Acute myocardial infarction NOS | 90861 | Transluminal operations on aneurysmal segment of aorta NOS |
| 2760 | Peripheral vascular disease NOS | 36717 | Cerebral infarction due to thrombosis of cerebral arteries |
| 7111 | Insertion of iliac artery stent | 36443 | Bypass iliac artery by femoral/femoral art anastomosis NEC |
| 3704 | Acute subendocardial infarction | 97661 | Replace aneurysm fem artery by fem/fem art anastomosis NEC |
| 12413 | Carotid artery doppler abnormal | 68385 | Other replacement aneurysmal femoral artery by anastomosis |
| 3530 | Peripheral vascular disease NOS | 40619 | Percutaneous transluminal embolisation of popliteal artery |
| 10504 | Right sided cerebral infarction | 48755 | Emerg bypass bifurc aorta by anast aorta to femoral artery |
| 5363 | CVA - cerebral artery occlusion | 17345 | AAA - Abdominal aortic aneurysm without mention of rupture |
| 39403 | Sequelae of cerebral infarction | 72491 | Other emerg bypass femoral or popliteal art by anastomosis |
| 18689 | Middle cerebral artery syndrome | 70446 | Endovascular stenting infrarenal abdominal aortic aneurysm |
| 32403 | Diabetes mellitus with gangrene | 23352 | Bypass aorta by anastomosis axillary to femoral artery NEC |
| 63396 | Reconstruction of femoral artery | 61670 | Diab mellit insulin-glucose infus acute myocardial infarct |
| 63830 | Stenosis of precerebral arteries | 71274 | Occlusion+stenosis of multiple and bilat cerebral arteries |
| 12733 | Carotid endarterectomy and patch | 50241 | Anterior spinal and vertebral artery compression syndromes |
| 20672 | Reconstruction of carotid artery | 55554 | Bypass leg artery by aorta/com femoral art anastomosis NEC |
| 1677 | MI - acute myocardial infarction | 60693 | Emerg bypass pop art by pop/pop art anast c vein graft NEC |
| 18842 | Subsequent myocardial infarction | 23671 | Cerebral infarct due to thrombosis of precerebral arteries |
| 38921 | Other bypass of iliac artery NOS | 48939 | Emerg bypass femoral artery by fem/fem art anastomosis NEC |
| 4917 | Evacuation of subdural haematoma | 65025 | Diabetes mellitus NOS with peripheral circulatory disorder |
| 71585 | Precerebral artery occlusion NOS | 72448 | Emerg bypass iliac artery by femoral/femoral art anast NEC |
| 61255 | Open embolectomy of iliac artery | 95573 | Emerg aortic bypass by anastomosis axillary to femoral art |
| 12331 | Other bypass of popliteal artery | 96838 | [X]Acute transmural myocardial infarction of unspecif site |
| 34117 | Other cerebrovascular disease OS | 59189 | Ruptur cardiac wall w'out haemopericard/cur comp fol ac MI |
| 29643 | Acute inferoposterior infarction | 66879 | Emerg bypass pop art by pop/tib art anast c vein graft NEC |
| 21118 | Vertebro-basilar artery syndrome | 53675 | Bypass femoral artery by fem/peron a anast c vein graft NEC |
| 38546 | Dacron graft operations on aorta | 28030 | Bypass femoral artery by fem/pop art anast c vein graft NEC |
| 34803 | Other acute myocardial infarction | 94069 | Endovas insertion of stent graft for aortic bifurcation NEC |
| 5943 | Other peripheral vascular disease | 27580 | Bypass femoral artery by fem/pop art anast c prosthesis NEC |
| 39546 | [X]Other forms of angina pectoris | 98565 | OS translum ins stent graft for aneurysmal segment of aorta |
| 48846 | Operation on popliteal artery NEC | 23708 | Atrial septal defect/curr comp folow acut myocardal infarct |
| 9759 | Leaking abdominal aortic aneurysm | 11039 | Excepted from stroke quality indicators: Patient unsuitable |
| 40098 | Suspected cerebrovascular disease | 56495 | Replace aneurysm bifurc aorta by anast aorta to femoral art |
| 37493 | Other cerebrovascular disease NOS | 28616 | Bypass iliac artery by iliac/femoral artery anastomosis NEC |
| 51166 | Y graft abdominal Aortic aneurysm | 24097 | Bypass popliteal artery by pop/pop a anast c vein graft NEC |
| 62626 | Acute papillary muscle infarction | 98175 | Endovascular insertion of stent for aorto-uniiliac aneurysm |
| 17734 | Subdural haematoma - nontraumatic | 37657 | Ventric septal defect/curr comp fol acut myocardal infarctn |
| 19280 | Anterior cerebral artery syndrome | 29553 | Thrombosis atrium,auric append&vent/curr comp foll acute MI |
| 66437 | Repair of common iliac artery NEC | 93134 | High-flow inter extrac intrac byp ext carot art mid cer art |
| 14898 | Lateral myocardial infarction NOS | 39877 | Bypass femoral artery by fem/tib art anast c prosthesis NEC |
| 39437 | Open embolectomy popliteal artery | 44250 | Emerg bypass iliac art by iliac/femoral art anastomosis NEC |
| 13572 | Ruptured abdominal aortic aneurysm | 24446 | Cerebral infarction due to embolism of precerebral arteries |
| 9119 | Open embolectomy of femoral artery | 66804 | Bypass leg artery by aorta/deep femoral art anastomosis NEC |
| 18687 | DNA - Did not attend stroke clinic | 94784 | Emergency replacement aneurysmal superficial femoral artery |
| 4107 | Evacuation of extradural haematoma | 41823 | Bypass femoral artery by fem/tib art anast c vein graft NEC |
| 51326 | Other precerebral artery occlusion | 59940 | Ruptur chordae tendinae/curr comp fol acute myocard infarct |
| 62866 | Reconstruction of iliac artery NOS | 95347 | [X]Other vascular syndroms/brain in cerebrovasculr diseases |
| 68366 | Reconstruction of vertebral artery | 67982 | Bypass femoral artery by fem/peron a anast c prosthesis NEC |
| 33430 | Operation on aneurysm of aorta NEC | 96699 | Emerg replace aneurysm iliac art by iliac/femoral art anast |
| 53810 | [X]Other intracerebral haemorrhage | 81445 | Percutaneous transluminal insertion of stent femoral artery |
| 14897 | Anterior myocardial infarction NOS | 32492 | Bypass common iliac artery by aorta/com iliac art anast NEC |
| 51211 | Endarterectomy of iliac artery NEC | 31218 | Stroke/transient ischaemic attack monitoring administration |
| 1678 | Inferior myocardial infarction NOS | 94556 | Other replacement of aneurysmal femoral/popliteal artery OS |
| 19260 | Posterior cerebral artery syndrome | 94331 | Translum insert stent graft for aneurysmal segment of aorta |
| 40347 | Open embolectomy of carotid artery | 64555 | Bypass popliteal artery by pop/pop a anast c prosthesis NEC |
| 28125 | Reconstruction of popliteal artery | 54192 | Emerg replace aneurysm abdom aorta by anast aorta/aorta NEC |
| 32272 | Postoperative myocardial infarction | 48700 | Bypass popliteal artery by pop/tib a anast c vein graft NEC |
| 16363 | Open thrombectomy of femoral artery | 63357 | Diabetes mellitus, adult, + peripheral circulatory disorder |
| 16991 | Iliac and femoral artery operations | 60465 | Bypass popliteal artery by pop/tib a anast c prosthesis NEC |
| 4273 | Subdural haemorrhage - nontraumatic | 40397 | Other open operations on femoral artery or popliteal artery |
| 43418 | Epidural haematoma following injury | 56510 | Replace aneurysm bifurc aorta by anast aorta to iliac artery |
| 40302 | Axillo-unifemoral PTFE bypass graft | 66232 | Emerg replace aneurysm infrarenal aorta by anast aorta/aorta |
| 23892 | Posterior myocardial infarction NOS | 52342 | Emerg bypass femoral art by fem/pop a anast c vein graft NEC |
| 36390 | Aneurysm of internal carotid artery | 96472 | Replace aneurysm fem art by fem/pop art anastom c prosth NEC |
| 17220 | Emergency repair of aortic aneurysm | 45428 | Bypass femoral artery by femoral/femoral art anastomosis NEC |
| 50678 | Aneurysm of external carotid artery | 57793 | Profundoplasty femoral artery & patch repair deep fem artery |
| 44765 | Carotid artery syndrome hemispheric | 68141 | Emerg bypass comm iliac art by aorta/com iliac art anast NEC |
| 63920 | Ruptured suprarenal aortic aneurysm | 94682 | Endovas insert of stent graft for suprarenal aortic aneurysm |
| 94351 | Open traumatic subdural haemorrhage | 62025 | Emerg replace aneurysm fem art by fem/pop anast c vein graft |
| 63467 | True posterior myocardial infarction | 62775 | Emerg bypass pop art by pop/peron art anast c vein graft NEC |
| 20657 | Revision of reconstruction of artery | 56458 | Ref to multidisciplinary stroke function improvement service |
| 60664 | Cardiac enzymes abnormal - first set | 9943 | Cereb autosom dominant arteriop subcort infarcts leukoenceph |
| 2654 | Endarterectomy of carotid artery NEC | 6960 | CVA - cerebrovascular accid due to intracerebral haemorrhage |
| 98188 | Small vessel cerebrovascular disease | 71141 | Replace aneurysm pop art by pop/pop a anast c vein graft NEC |
| 34037 | Open insertion of iliac artery stent | 91627 | [X]Cerebrl infarctn due/unspcf occlusn or sten/cerebrl artrs |
| 47835 | Profundoplasty of femoral artery NEC | 19996 | Replace aneurysm abdominal aorta by anast aorta to aorta NEC |
| 31053 | [D]Widespread diabetic foot gangrene | 44553 | Replace aneurys infrarenal aorta by anast aorta to aorta NEC |
| 36952 | Other bypass of bifurcation of aorta | 96656 | Other replacement of aneurysmal femoral/popliteal artery NOS |
| 97003 | Reconstruction of carotid artery NOS | 55476 | Emerg replace aneurysm fem art by fem/pop art anast c prosth |
| 18816 | Endarterectomy of femoral artery NEC | 67818 | Emerg bypass popliteal art by pop/pop art anast c prosth NEC |
| 55351 | Delivery of rehabilitation for stroke | 31704 | Occlusion/stenosis cerebral arts not result cerebral infarct |
| 6569 | Subdural haemorrhage following injury | 89913 | Stroke/transient ischaemic attack monitoring telephone invte |
| 43449 | Intracranial bypass to carotid artery | 70922 | Emerg bypass popliteal artery by pop/fem art anastomosis NEC |
| 46017 | Other acute myocardial infarction NOS | 66917 | Emerg bypass leg artery by aorta/com fem art anastomosis NEC |
| 36178 | Extradural haemorrhage - nontraumatic | 93770 | Percutaneous transluminal insertion of stent cerebral artery |
| 22008 | Vertebral artery compression syndrome | 89365 | Byp carot art anastom superfic tempor artery middle cere art |
| 5702 | Peripheral ischaemic vascular disease | 58092 | Replace aneurysm pop art by pop/pop art anastom c prosth NEC |
| 73471 | Open traumatic extradural haemorrhage | 46112 | Postoperative transmural myocardial infarction anterior wall |
| 23871 | Peripheral angiopathic disease EC NOS | 69474 | Rupture papillary muscle/curr comp fol acute myocard infarct |
| 68069 | Endovascular repair of carotid artery | 89470 | Emerg replace aneurysm pop art by pop/tib anast c vein graft |
| 37787 | Other bypass of common femoral artery | 40787 | Thoracoabdominal aortic aneurysm, without mention of rupture |
| 63280 | Reconstruction of common iliac artery | 51465 | Stroke/transient ischaemic attack monitoring verbal invitati |
| 42645 | Open thrombectomy of popliteal artery | 53980 | Traumatic subdural haematoma without open intracranial wound |
| 2883 | Closed traumatic subdural haemorrhage | 9099 | Other emergency bypass of femoral artery or popliteal artery |
| 98204 | [SO]Branch of external carotid artery | 61666 | Emergency replacement of aneurysmal femoral/popliteal artery |
| 48149 | Sequelae of intracerebral haemorrhage | 96255 | Emerg bypass femoral art by fem/tib a anast c vein graft NEC |
| 51331 | Other open operations on iliac artery | 56007 | Subarachnoid haemorrhage from carotid siphon and bifurcation |
| 5682 | Cerebral haemorrhage following injury | 36423 | Certain current complication follow acute myocardial infarct |
| 59756 | Replacement of aneurysmal iliac artery | 33650 | Percut transluminal coronary thrombolysis with streptokinase |
| 65669 | Profundoplasty of popliteal artery NEC | 70448 | Diabetes mellitus, juvenile +peripheral circulatory disorder |
| 68906 | Endarterectomy of vertebral artery NEC | 46276 | Postoperative transmural myocardial infarction inferior wall |
| 12736 | Type 2 diabetes mellitus with gangrene | 95416 | Replace aneurysm popliteal artery by pop/fem anastomosis NEC |
| 46150 | Type 2 diabetes mellitus with gangrene | 93627 | Translum ins stent graft for aneurysmal segment of aorta NOS |
| 36136 | Endarterectomy of popliteal artery NEC | 55394 | Emerg replace aneurysm pop artery by pop/fem art anastomosis |
| 33807 | Diabetes mellitus, adult with gangrene | 83577 | Endovas ins stent graft for infrarenal abdom aortic aneurysm |
| 69993 | Type 1 diabetes mellitus with gangrene | 97217 | Endovascul insert stent infrarenal abdominal aortic aneurysm |
| 70536 | Acute cerebrovascular insufficiency NOS | 40996 | Percut translum coronary thrombolytic therapy- streptokinase |
| 43089 | Uncomplicated arteriosclerotic dementia | 68412 | Bypass popliteal art by pop/peron art anast c vein graft NEC |
| 18604 | Stroke due to intracerebral haemorrhage | 54379 | Replace aneurys suprarenal aorta by anast aorta to aorta NEC |
| 45421 | Closed traumatic extradural haemorrhage | 57183 | Mitochond encephalopathy, lact acidosis & strokelike episode |
| 51311 | Other specified cerebrovascular disease | 72448 | Emerg bypass iliac artery by femoral/femoral art anast NEC |
| 26232 | Tube graft of Abdominal aortic aneurysm | 89470 | Emerg replace aneurysm pop art by pop/tib anast c vein graft |
| 56912 | Arteriosclerotic dementia with delirium | 23352 | Bypass aorta by anastomosis axillary to femoral artery NEC |
| 55467 | Arteriosclerotic dementia with paranoia | 94556 | Other replacement of aneurysmal femoral/popliteal artery OS |
