# Supplementary material for: Statin use in cancer survivors versus the general population: cohort study using primary care data from the UK clinical practice research datalink
Source: BMC Cancer. 2018 Oct 22;18:1018. doi: 10.1186/s12885-018-4947-8 (PMC6196462; doi:10.1186/s12885-018-4947-8)
Supplement: Supplementary file 3 — Table S3. Code list for the identification of a cardiovascular risk event recorded in the CPRD database. (DOCX 20 kb) [file 12885_2018_4947_MOESM3_ESM.docx]

**Table S3: Code list for the identification of a cardiovascular risk score recorded in the CPRD database**

| **medcode** | **readterm** | **score_type** | **risk_level** |
| --- | --- | --- | --- |
| 7913 | Coronary heart disease risk | Coronary risk score | No information from Read term |
| 10128 | Cardiovascular event risk | Cardiovascular risk score | No information from Read term |
| 10302 | Framingham coronary heart disease 10 year risk score | Coronary risk score | No information from Read term |
| 13283 | Coronary heart disease risk | Coronary risk score | No information from Read term |
| 18581 | Low risk of primary heart disease | Cardiovascular risk score | No information from Read term |
| 18948 | Moderate risk of primary heart disease | Cardiovascular risk score | No information from Read term |
| 22210 | High risk of primary heart disease | Cardiovascular risk score | No information from Read term |
| 24721 | Framingham coronary heart disease 10 year risk score | Coronary risk score | No information from Read term |
| 26627 | At risk of heart disease | Cardiovascular risk score | No information from Read term |
| 29433 | High risk of heart disease | Cardiovascular risk score | No information from Read term |
| 36908 | UKPDS 10yr coronary heart disease risk score | Coronary risk score | No information from Read term |
| 43934 | Joint British Societies cardiac risk score | Cardiovascular risk score | No information from Read term |
| 43938 | Framingham coronary heart disease 10 yr adjusted risk score | Coronary risk score | No information from Read term |
| 55103 | JBS cardiovascular disease risk 10-20% over next 10 years | Cardiovascular risk score | Read term cat 10-20 |
| 55104 | JBS cardiovascular disease risk <10% over next 10 years | Cardiovascular risk score | Read term cat <10 |
| 55105 | JBS cardiovascular disease risk >30% over next 10 years | Cardiovascular risk score | Read term cat >30 |
| 55109 | JBS cardiovascular disease risk >20% up to 30% ov next 10 yr | Cardiovascular risk score | Read term cat >20 & <=30 |
| 71748 | Coronary heart disease risk clinical management plan | Coronary risk score | No information from Read term |
| 85854 | Review of patient at risk from coronary heart disease | Coronary risk score | No information from Read term |
| 95889 | Assessing cardiovascular risk using SIGN score | Cardiovascular risk score | No information from Read term |
| 95948 | QRISK cardiovascular disease 10 year risk score | Cardiovascular risk score | No information from Read term |
| 96275 | ASSIGN score | Cardiovascular risk score | No information from Read term |
| 96886 | Cardiovascular disease risk assessment done | Cardiovascular risk score | No information from Read term |
| 96899 | Cardiovascular disease risk assessment indicated | Cardiovascular risk score | No information from Read term |
| 97641 | Cardiovascular disease risk assessment | Cardiovascular risk score | No information from Read term |
| 98113 | QRISK2 cardiovascular disease 10 year risk score | Cardiovascular risk score | No information from Read term |
| 98120 | Framingham 1991 cardiovascular disease 10 year risk score | Cardiovascular risk score | No information from Read term |
| 98429 | Cardiovascular disease high risk review | Cardiovascular risk score | No information from Read term |
| 100937 | CVD (cardiovascular disease) risk assessment by third party | Cardiovascular risk score | No information from Read term |
| 101644 | Consent given for cardiovascular health risk assessment | Cardiovascular risk score | No information from Read term |
| 104175 | Joint British Societies cardiovascular disease risk score | Cardiovascular risk score | No information from Read term |
| 105223 | At risk of cardiovascular disease | Cardiovascular risk score | No information from Read term |
| 105901 | High risk of cardiovascular disease | Cardiovascular risk score | No information from Read term |
