# Supplementary material for: Statin use in cancer survivors versus the general population: cohort study using primary care data from the UK clinical practice research datalink
Source: BMC Cancer. 2018 Oct 22;18:1018. doi: 10.1186/s12885-018-4947-8 (PMC6196462; doi:10.1186/s12885-018-4947-8)
Supplement: Supplementary file 4 — Table S4. Product codes for the identification of statin therapy. (DOCX 26 kb) [file 12885_2018_4947_MOESM4_ESM.docx]

**Table S4: Product codes for the identification of statin therapy**

| **prodcode** | **Substance** | **prodcode** | **Substance** | **prodcode** | **Substance** |
| --- | --- | --- | --- | --- | --- |
| 47065 | atorvastatin calcium | 1219 | pravastatin sodium | 802 | simvastatin |
| 47090 | atorvastatin calcium | 54607 | pravastatin sodium | 54947 | simvastatin |
| 47630 | atorvastatin calcium | 50925 | pravastatin sodium | 54985 | simvastatin |
| 57348 | atorvastatin calcium trihydrate | 51890 | pravastatin sodium | 55452 | simvastatin |
| 28 | atorvastatin calcium trihydrate | 3690 | pravastatin sodium | 50483 | simvastatin |
| 56248 | atorvastatin calcium trihydrate | 54435 | pravastatin sodium | 54266 | simvastatin |
| 75 | atorvastatin calcium trihydrate | 56893 | pravastatin sodium | 54976 | simvastatin |
| 55444 | atorvastatin calcium trihydrate | 490 | pravastatin sodium | 48051 | simvastatin |
| 51622 | atorvastatin calcium trihydrate | 40382 | pravastatin sodium | 52953 | simvastatin |
| 58394 | atorvastatin calcium trihydrate | 56607 | pravastatin sodium | 58315 | simvastatin |
| 51359 | atorvastatin calcium trihydrate | 56735 | pravastatin sodium | 45219 | simvastatin |
| 50236 | atorvastatin calcium trihydrate | 56916 | pravastatin sodium | 40601 | simvastatin |
| 58110 | atorvastatin calcium trihydrate | 1221 | pravastatin sodium | 53822 | simvastatin |
| 50272 | atorvastatin calcium trihydrate | 57397 | pravastatin sodium | 49587 | simvastatin |
| 7374 | atorvastatin calcium trihydrate | 57108 | pravastatin sodium | 52625 | simvastatin |
| 52821 | atorvastatin calcium trihydrate | 34820 | pravastatin sodium | 39060 | simvastatin |
| 57117 | atorvastatin calcium trihydrate | 57296 | pravastatin sodium | 48867 | simvastatin |
| 55727 | atorvastatin calcium trihydrate | 51676 | pravastatin sodium | 34381 | simvastatin |
| 58418 | atorvastatin calcium trihydrate | 57137 | pravastatin sodium | 34316 | simvastatin |
| 52460 | atorvastatin calcium trihydrate | 1223 | pravastatin sodium | 32909 | simvastatin |
| 53890 | atorvastatin calcium trihydrate | 43218 | pravastatin sodium | 13041 | simvastatin |
| 2955 | atorvastatin calcium trihydrate | 730 | pravastatin sodium | 53340 | simvastatin |
| 17683 | atorvastatin calcium trihydrate | 47988 | pravastatin sodium | 50882 | simvastatin |
| 56841 | atorvastatin calcium trihydrate | 52755 | pravastatin sodium | 34376 | simvastatin |
| 52397 | atorvastatin calcium trihydrate | 56146 | pravastatin sodium | 45235 | simvastatin |
| 52398 | atorvastatin calcium trihydrate | 55912 | pravastatin sodium | 34891 | simvastatin |
| 49558 | atorvastatin calcium trihydrate | 32921 | pravastatin sodium | 52098 | simvastatin |
| 53594 | atorvastatin calcium trihydrate | 36377 | pravastatin sodium | 48078 | simvastatin |
| 3411 | atorvastatin calcium trihydrate | 48097 | pravastatin sodium | 34969 | simvastatin |
| 47721 | atorvastatin calcium trihydrate | 9897 | rosuvastatin calcium | 34955 | simvastatin |
| 55034 | atorvastatin calcium trihydrate | 17688 | rosuvastatin calcium | 34907 | simvastatin |
| 52168 | atorvastatin calcium trihydrate | 57763 | rosuvastatin calcium | 50754 | simvastatin |
| 50788 | atorvastatin calcium trihydrate | 57999 | rosuvastatin calcium | 44878 | simvastatin |
| 53887 | atorvastatin calcium trihydrate | 15252 | rosuvastatin calcium | 34746 | simvastatin |
| 51134 | atorvastatin calcium trihydrate | 7554 | rosuvastatin calcium | 50564 | simvastatin |
| 51200 | atorvastatin calcium trihydrate | 9930 | rosuvastatin calcium | 34535 | simvastatin |
| 57834 | atorvastatin calcium trihydrate | 7347 | rosuvastatin calcium | 34502 | simvastatin |
| 50963 | atorvastatin calcium trihydrate | 53460 | rosuvastatin calcium | 54655 | simvastatin |
| 57836 | atorvastatin calcium trihydrate | 6213 | rosuvastatin calcium | 34545 | simvastatin |
| 58041 | atorvastatin calcium trihydrate | 713 | rosuvastatin calcium | 46956 | simvastatin |
| 51876 | atorvastatin calcium trihydrate | 11815 | simvastatin | 53908 | simvastatin |
| 54535 | atorvastatin calcium trihydrate | 10206 | simvastatin | 33082 | simvastatin |
| 52211 | atorvastatin calcium trihydrate | 10183 | simvastatin | 34879 | simvastatin |
| 48518 | atorvastatin calcium trihydrate | 47948 | simvastatin | 7196 | simvastatin |
| 53772 | atorvastatin calcium trihydrate | 56481 | simvastatin | 52812 | simvastatin |
| 745 | atorvastatin calcium trihydrate | 48018 | simvastatin | 22579 | simvastatin |
| 56182 | atorvastatin calcium trihydrate | 34476 | simvastatin | 44528 | simvastatin |
| 5775 | atorvastatin calcium trihydrate | 25 | simvastatin | 34814 | simvastatin |
| 49751 | atorvastatin calcium trihydrate | 39870 | simvastatin | 40340 | simvastatin |
| 50790 | atorvastatin calcium trihydrate | 51233 | simvastatin | 51715 | simvastatin |
| 52097 | atorvastatin calcium trihydrate | 57568 | simvastatin | 6168 | simvastatin |
| 56564 | atorvastatin calcium trihydrate | 51166 | simvastatin | 46878 | simvastatin |
| 52459 | atorvastatin calcium trihydrate | 51085 | simvastatin | 42 | simvastatin |
| 55032 | atorvastatin calcium trihydrate | 48431 | simvastatin | 31930 | simvastatin |
| 53813 | cerivastatin sodium | 34366 | simvastatin | 34312 | simvastatin |
| 5009 | cerivastatin sodium | 9920 | simvastatin | 45245 | simvastatin |
| 420 | cerivastatin sodium | 47774 | simvastatin | 53966 | simvastatin |
| 5251 | cerivastatin sodium | 50703 | simvastatin | 54819 | simvastatin |
| 31658 | cerivastatin sodium | 52257 | simvastatin | 41657 | simvastatin |
| 58480 | cerivastatin sodium | 52962 | simvastatin | 50670 | simvastatin |
| 55207 | cerivastatin sodium | 53087 | simvastatin | 2718 | simvastatin |
| 5278 | cerivastatin sodium | 56494 | simvastatin | 37434 | simvastatin |
| 18442 | cerivastatin sodium | 53415 | simvastatin | 45346 | simvastatin |
| 9315 | cerivastatin sodium | 54240 | simvastatin | 39652 | simvastatin |
| 4961 | cerivastatin sodium | 48058 | simvastatin | 44650 | simvastatin |
| 9316 | cerivastatin sodium | 52676 | simvastatin | 51483 | simvastatin |
| 16186 | ezetimibe/simvastatin | 34353 | simvastatin | 34481 | simvastatin |
| 14219 | ezetimibe/simvastatin | 818 | simvastatin | 49062 | simvastatin |
| 53770 | fluvastatin sodium | 54493 | simvastatin | 51 | simvastatin |
| 2137 | fluvastatin sodium | 39675 | simvastatin | 5148 | simvastatin |
| 5985 | fluvastatin sodium | 49061 | simvastatin | 10172 | simvastatin/ezetimibe |
| 9153 | fluvastatin sodium | 34560 | simvastatin | 17059 | simvastatin/ezetimibe |
| 11627 | fluvastatin sodium | 53676 | simvastatin | 7552 | simvastatin/ezetimibe |
| 8380 | fluvastatin sodium |  |  | 21020 | simvastatin/ezetimibe |
| 379 | fluvastatin sodium |  |  |  |  |
|  |  |  |  | 54606 |  |
|  |  |  |  | 57329 |  |
|  |  |  |  | 56165 |  |
|  |  |  |  | 54992 |  |
|  |  |  |  | 56065 |  |
|  |  |  |  | 56097 |  |
|  |  |  |  | 48973 |  |
|  |  |  |  | 56016 |  |
|  |  |  |  | 48346 |  |
|  |  |  |  | 48221 |  |
|  |  |  |  | 58617 |  |
