# Supplementary material for: Statin use in cancer survivors versus the general population: cohort study using primary care data from the UK clinical practice research datalink
Source: BMC Cancer. 2018 Oct 22;18:1018. doi: 10.1186/s12885-018-4947-8 (PMC6196462; doi:10.1186/s12885-018-4947-8)
Supplement: Supplementary file 5 — Table S5. Proportion (%) of individuals with a blood pressure, cholesterol or cardiovascular risk score recorded in the past 5 years, by age and gender. (DOCX 20 kb) [file 12885_2018_4947_MOESM5_ESM.docx]

**Table S5: Proportion (%) of individuals with a blood pressure, cholesterol or cardiovascular risk score recorded in the past 5 years, by age and gender**

|  | **Blood pressure recorded** | | **Cholesterol recorded** | | **CVR score recorded** | |
| --- | --- | --- | --- | --- | --- | --- |
|  | **Cancer survivors** | **Controls** | **Cancer survivors** | **Controls** | **Cancer survivors** | **Controls** |
| Age (yrs) and sex |  |  |  |  |  |  |
| **45** |  |  |  |  |  |  |
| **Males** | 70.40 | 59.92 | 40.46 | 32.61 | 8.99 | 6.76 |
| **Females** | 84.97 | 81.47 | 33.20 | 33.95 | 5.57 | 6.41 |
| **50** |  |  |  |  |  |  |
| **Males** | 85.64 | 72.72 | 51.17 | 42.71 | 10.06 | 9.79 |
| **Females** | 90.48 | 88.30 | 42.18 | 43.87 | 10.01 | 9.46 |
| **55** |  |  |  |  |  |  |
| **Males** | 89.60 | 77.26 | 61.44 | 51.54 | 14.92 | 12.42 |
| **Females** | 92.06 | 89.44 | 50.55 | 51.82 | 12.45 | 12.11 |
| **60** |  |  |  |  |  |  |
| **Males** | 92.35 | 81.48 | 67.70 | 58.52 | 17.92 | 14.44 |
| **Females** | 92.44 | 90.37 | 58.08 | 58.46 | 14.46 | 14.08 |
| **65** |  |  |  |  |  |  |
| **Males** | 94.40 | 86.31 | 74.43 | 67.52 | 19.98 | 18.07 |
| **Females** | 94.76 | 91.50 | 67.40 | 66.49 | 17.70 | 17.43 |
| **70** |  |  |  |  |  |  |
| **Males** | 96.39 | 90.26 | 78.38 | 72.33 | 21.26 | 18.32 |
| **Females** | 95.52 | 92.61 | 72.58 | 71.25 | 18.83 | 17.54 |
| **75** |  |  |  |  |  |  |
| **Males** | 97.16 | 91.62 | 80.10 | 73.96 | 18.50 | 16.94 |
| **Females** | 96.84 | 93.92 | 76.18 | 73.47 | 19.22 | 16.68 |
