# Supplementary material for: Statin use in cancer survivors versus the general population: cohort study using primary care data from the UK clinical practice research datalink
Source: BMC Cancer. 2018 Oct 22;18:1018. doi: 10.1186/s12885-018-4947-8 (PMC6196462; doi:10.1186/s12885-018-4947-8)
Supplement: Supplementary file 6 — ISAC protocol 16_163. (PDF 735 kb) [file 12885_2018_4947_MOESM6_ESM.pdf]

ISAC protocol 16_163
Cohort study on the uptake of pharmacological treatments to prevent cardiovascular diseases in UK cancer survivors versus general population from 2005-2013.

Note: The analysis completed for this manuscript (Chidwick et al., 2018) focuses on specific aims 3 and 4. Statin use for primary prevention of cardiovascular disease was investigated and not secondary prevention or use of anti-hypertensive drugs. This analysis follows the protocol closely with the following exceptions:

- For specific aim 2, the sensitivity analysis counting statin prescription within 3 months of the first high CV risk score was not completed.
- The following covariates were not considered for either aim: cholesterol / HDL ratio, other risk factors (family history of CVD, ethnicity etc), type of cancer.

Simple analyses relating to specific aim 1 were completed to support aims 3 and 4. These are described as exploratory in the manuscript.

A. Lay Summary (Max. 200 words)

Half of all cancer patients are now expected to survive from their cancer for 10 years. The risk of developing cardiovascular disease (i.e. having a heart attack or stroke) is recognised as a key potential life-long consequence of cancer treatment. Cardiovascular disease is the second leading cause of long-term complications and death among cancer survivors, after secondary cancers. Guidelines recommend people aged 40-74 years should have their blood pressure, cholesterol, and other risk factors measured every 5 years, and their risk of developing cardiovascular disease in the next 10 years should be estimated. Those patients considered at high risk should be offered advice to lower their risk and should typically be offered cholesterol lowering therapy (a statin) and a medicine to lower blood pressure. It is not clear whether these cardiovascular risk prevention guidelines are being followed.

With this study using UK primary care data (2005-2013) we aim to understand whether recommended risk factor monitoring and medicines to prevent CVD are optimally implemented among cancer survivors compared to the general population. We will also investigate whether cancer survivors are more or less likely to continue or stop taking medicines for CVD risk versus the general population.

B. Technical Summary (Max. 200 words)

This retrospective cohort study in Clinical Practice Research Datalink (2005-2013) aims to understand whether recommended risk factor monitoring (blood pressure, cholesterol, cardiovascular disease risk scores) and pharmacological interventions (statins and antihypertensives) to prevent cardiovascular disease are optimally implemented among cancer survivors versus the general population; and whether persistence on preventive therapies is different in cancer survivors.

The study population will be drawn from all research quality patients in CPRD who were 40 years or older between 2005 and 2013. Follow-up time will be categorised as non-cancer, first year since cancer diagnosis, and ≥1 year since cancer diagnosis (i.e. cancer survivors). Only patients with a cardiovascular risk score recorded will be

included in the statin uptake analysis and those who initiate a statin the persistence analysis.

Descriptive statistics will be used to describe baseline demographics and cardiovascular risk distribution of the cancer survivors cross-tabulated with controls. Logistic regression modelling will be used to produce unadjusted and adjusted ORs, to estimate the association between cancer survivorship and initiation of therapy among those with high recorded cardiovascular risk, adjusted for confounders. We will measure time-to-discontinuation of therapy using Kaplein Meier curves. Hazard ratios for persistence will be estimated using Cox proportional hazards model.

C. Objectives, Specific Aims and Rationale

(i) **Objectives:** We aim to understand whether recommended risk factor monitoring and pharmacological interventions to prevent cardiovascular disease (i.e. prescription of preventive drugs for blood pressure and lipid control) are optimally implemented among cancer survivors. We also aim to understand whether persistence on cardiovascular disease (CVD) preventive therapies is different in cancer survivors compared with the general population by assessing whether cancer survivors who commence therapy with a statin or antihypertensive (AHT) have longer or shorter time to first drug discontinuation versus non-cancer controls. Predictors of short persistence will also be assessed.

(ii) **Specific Aims:**
1: To describe the monitoring of blood pressure and lipid levels, and recording of CVD risk scores among cancer survivors and controls with no cancer diagnosis. Proportions of patients with a measurement (blood pressure/lipids/CVD risk score) recorded in the past 1, 3, and 5 years will be presented according to a) sex, b) age range, and c) time since cancer diagnosis.

2: To describe the overall distribution of cardiovascular risk by age and sex, in terms of blood pressure, lipids, CVD risk score, among cancer survivors and non-cancer controls, subject to adequate recording as explored in Objective 1.

3: To investigate whether there are differences in the extent to which recommendations for pharmacological cardiovascular disease prevention according to calculated risk are being followed in cancer survivors compared with the general population.

Research Question: Does a cancer history/diagnosis change the likelihood of being prescribed a statin or AHT for primary or secondary prevention among adults >40 years attending primary care practices in the UK? How do the proposed associations change according to time since cancer diagnosis (e.g. <5, >5 years since cancer diagnosis)?

Null hypothesis 1: Among people with an indication for CVD preventive therapy with a statin (i.e. those with a CVD risk score >20%) there is no difference in the proportion who initiate a statin within one month of the high CVD risk record between cancer survivors and non-cancer controls (OR=1)

Null hypothesis 2: Among people with an indication for CVD preventive therapy with an AHT, there is no difference in the proportion who initiate an AHT within one month of becoming indicated (i.e. CVD risk score >20% and Stage 1 hypertension [BP ≥140/90mmHg] OR Stage 2 Hypertension [≥160/100mmHg] regardless of CVD risk score) between cancer survivors and non-cancer controls (OR=1)

4: To investigate whether persistence on CVD preventive therapies is different in cancer survivors compared with the general population we will determine whether cancer survivors who commence therapy with a statin or AHT have longer or shorter time to first drug discontinuation versus non-cancer controls. Predictors of short persistence will also be assessed.

Research Question: Does a cancer history/diagnosis change the likelihood of remaining on statin or antihypertensive therapy (AHT) for primary or secondary prevention amongst adults >40 years attending primary care practices in the UK? How do the proposed associations change according to time since cancer diagnosis (e.g. <5, >5 years since cancer diagnosis)?

Null hypothesis 3: After commencing therapy with a statin the time to first drug discontinuation is no different in cancer survivors versus non-cancer controls

Null hypothesis 4: After commencing therapy with an AHT the time to first drug discontinuation is no different in cancer survivors versus non-cancer controls

(iii) **Rationale**

To inform prevention initiatives among cancer survivors, it is necessary to understand whether current cardiovascular risk prevention guidelines are being implemented in this population and whether patients adhere to preventive therapies.

D. Background

Half of all cancer patients are now expected to survive from their cancer for at least 10 years.[1,2] Consequently, the population of cancer survivors (i.e. people living with a previous cancer diagnosis) is increasing, standing at 2 million in the UK,[3] 14 million in the US,[4] and tens of millions worldwide. The long-term health of these individuals is of increasing concern.[5] The potential consequences of cancer and its treatment are wide-ranging,[6] and the need for long-term interdisciplinary monitoring and care of cancer survivors is an increasing priority. However, the field is relatively new, and the epidemiological evidence base needed to guide policy is limited.[7,8]

Cardiovascular disease (CVD) risk is increasingly recognised as a key potential life-long consequence of cancer treatment, as illustrated by the recent emergence of cardio-oncology as a recognised field.[9] CVD is the second leading cause of long-term morbidity and death among cancer survivors, after second malignancies.[10]

In the years following diagnosis, an absence of specific guidelines for cardiovascular risk management means cancer survivors are likely to fall under broader guidelines for long-term prevention, the cornerstones of which are lipid and blood pressure management with statins and antihypertensive drugs, and minimisation of lifestyle-

associated risk factors.[11] However, it is not clear whether a diagnosis or history of cancer compromises implementation of these risk prevention strategies.

Since 2005 guidelines on cardiovascular disease prevention have recommended all those patients with CVD risk ≥20% over 10 years (i.e. high risk) are offered statin therapy.[12][13] NICE guidelines released in 2014 changed the recommendation to all those patients with CVD risk ≥10% over 10 years, substantially increasing the number of patients indicated for therapy with a statin. Guidelines for primary prevention of CVD in the UK are described in Appendix 1.

The NHS Health Checks (which include measuring blood pressure, and cholesterol and absolute cardiovascular risk) recently were rolled out across the UK in 2009 to prevent heart disease, stroke, kidney disease, type 2 diabetes or certain types of dementia in 40-74 year olds. NHS Health Checks are recommended every 5 years.[14]

This study aims to understand whether cardiovascular risk prevention guidelines are being implemented appropriately in cancer survivors.

E. Study Type

Descriptive and hypothesis testing

F. Study Design

Longitudinal population based open cohort study.

G. Sample Size

Objective 1 and 2

We estimate there are approximately 4.17 million patients in the CPRD database over the age of 40 during the study time period who are eligible for this study. To facilitate data management while still producing sufficiently precise estimates of descriptive statistics, we will use a random sample of 1 million eligible patients only for objectives 1 and 2. Our feasibility work suggests that confidence interval widths will still be narrow (± 2-3.5%) for our completeness statistics with this reduced dataset. The random sample will be selected by sorting all eligible patients on a pseudo-random number generated by Stata, and taking the first 1 million of the resultant patient ids.

Objective 3

Based on a preliminary feasibility analysis looking at statin uptake in a sample from the CPRD database we found that 23% of patients in the non-cancer cohort (i.e. the general population) received a statin within 1 month after their high CVD risk score being recorded, in concordance with an earlier study.[15] With an estimated 115,089 patients in the non-cancer cohort and 6,695 in the cancer survivor cohort based on our feasibility work (Table 1) we will have 80% power at 5% significance to detect an odds ratio of ≤ 0.9185.

Table 1: All patients who meet study criteria and have a high CVD risk score recorded during the study time period*.

Patient cohort	CPRD sample dataset – 1 million patients [Jan 2014 build] (n)	Projected frequency for the entire CPRD database (n x13)
Non-cancer	8,853	115,089
1 yr since cancer	92	1,196
Survivor (>1yr)	515	6,695
Total	9,460	122,980

**Patients were excluded if they started a statin before their high CVD risk score.*

Objective 4

A study published by Vinogradova Y et al (2016)[16] of incident statin users identified in the CPRD database between 2002 and 2013 found the 47% of patients prescribed statin as primary prevention discontinued treatment.

From feasibility work, there will be approximately 568,165 eligible patients who commenced therapy with a statin during the study time period, with 8% are in the cancer survivor group. With 90% power and 5% alpha, and assuming 47% discontinue, this will allow us to detect a hazard ratio of 1.02 or 0.98 for cancer survivors vs controls, based on a Cox regression analysis (calculated using stpower cox in Stata).

H. Data Linkage Required (if applicable)

We expect socioeconomic status to be a confounder in the association between cancer and initiation of statins and antihypertensives therefore we are requesting index of multiple deprivation (IMD) 2010 quintiles. We plan to use practice level IMD data for the main analysis to preserve the study power, and the more granular patient-postcode level IMD in a sensitivity analysis among linked patients.

I. Study Population

Time:

The study time period will be from 1st January 2005 to 31st December 2013. All person-time will be categorised into "no-cancer" (includes pre-cancer and never-cancer follow-up time), "first year post-cancer diagnosis" and "≥1 year post cancer diagnosis". The third category of person-time defines the "cancer survivors".

Person:

Objective 1 & 2

We will include patients who were 40 years or older, between 1st January 2005 and 31st December 2012 and have at least 12 months of up to standard (UTS) CPRD follow-up prior to joining the cohort; 1 million eligible patients will be selected as described in section G.

Objective 3

We will include patients who were a) 40 years or older, between 1st January 2005 and 31st December 2012, b) have at least 12 months of up to standard (UTS) CPRD follow-up prior to joining the cohort and c) have their first high CVD risk score (>20%) ever (the index date) during their study follow-up time.

Patients will be excluded if they commenced therapy with a statin/AHT prior to the first high CVD risk score

Objective 4
We will include patients who were a) 40 years or older, between 1st January 2005 and 31st December 2012, b) have at least 12 months of up to standard (UTS) CPRD follow-up prior to joining the cohort and c) initiate therapy with a statin/AHT for the first time (i.e. incident users) during the study time period. Incident users must have at least 12 months of UTS follow-up in CPRD prior to first prescription, and date of therapy initiation is the index date.

Place: UK research quality CPRD practices (not restricted to practices that are eligible for linkage).

J. Selection of comparison group(s) or controls

Controls will be drawn from person-time categorised as "non-cancer" which includes person-time before a cancer diagnosis.

K. Exposures, Outcomes and Covariates

The **exposure** of interest is cancer survivorship (i.e. surviving at least 1 year after an incident cancer diagnosis). To identify cancer patients we will use NHS Read code lists developed by Krishnan Bhaskaran for a previous study;[17] their validity is suggested by analysis done by his doctoral student, who compared their observed incidences with national published incidence rates for common cancers (Appendix 2 Figure A1). The strategy for identifying the cancer codes in CPRD (Clinical and Referral files) is described in Appendix 3. Cancers recorded soon after a patient's UTS follow-up begins could reflect pre-existing or historical disease therefore patients will be excluded if their code occurs within 12 months after registration with the practice.
Patients will be considered at high cardiovascular risk and therefore eligible for statin therapy if they have a Read code/ Entity type indicating CVD risk ≥20% over 10 years in the clinical file, or additional clinical details file. Patients will be considered eligible for AHT therapy if CVD risk ≥20% and blood pressure equal to or over 140/90mmHg (stage 1 hypertension) OR Stage 2 Hypertension [≥160/100mmHg] regardless of CVD risk score ,

Patients recommended for secondary prevention will also be identified, based on having had a previous cardiovascular event, defined as coronary heart disease (myocardial infarction, angina and revascularisation procedures), cerebrovascular disease (stroke, transient ischaemic attack), or peripheral vascular disease (abdominal aortic aneurism and intermittent claudication). Codes relating to incident events and pre-existing conditions will be identified, based on codelists developed for the CALIBER project (Cardiovascular Disease Research Using Linked Bespoke Studies and Electronic Records, https://www.caliberresearch.org/).

Outcomes (descriptive)
Objective 1:
According to age, gender and year since cancer diagnosis, , we will describe:
- BP, lipids, CVD risk score recorded in the past 1,3 and 5 years

Medcodes/ Read terms indicating a CVD risk score was recorded are included in Appendix 4

Objective 2:
Of the patients with a measurement recorded, we will describe the following markers of cardiovascular risk at index date, according to age and gender:

- proportion with systolic BP >140 mm/Hg and >160mm/HG
- proportion with cholesterol >6mmol/L and >8mmol/L
- proportion with predicted 10 year risk of developing CVD >10%/ >20% / >30%.
- mean (SD) systolic blood pressure
- mean (SD) total cholesterol
- mean (SD) 10 year CV risk

Outcomes (hypothesis testing)

Objective 3:

- first prescription of statin therapy (see code list in Appendix 5) recorded within 1 month (and 3 months in sensitivity analysis) of first high CV risk record
- first prescription of AHT recorded within 1 month (and 3 months in sensitivity analysis) of first high CV risk record
- time from eligibility to first initiation of a statin/AHT

Objective 4:

- Time from statin initiation to first cessation of therapy
- Time from AHT initiation to first cessation of therapy

If there are no prescriptions within 90 days after the expected end date of a prescription, a patient will be defined as having ceased therapy. The date of cessation will be the expected end date of therapy, based on the prescription duration.

Covariates
Age, gender, practice, calendar time periods, BMI, alcohol use, smoking status, socioeconomic status, 10 year risk of developing CVD, Systolic BP, cholesterol , cholesterol/HDL ratio, previous CVD, other risks factors (family history of CVD, ethnicity etc), comorbidities (DM, CKD), type of cancer.

L. Data/ Statistical analysis

Data management and analyses will be undertaken using STATA® 13 statistical software. Start of patient follow-up will commence at the latest of 1/1/2005, patient's 40th birthday, 12 months up-to-standard follow-up since joining CPRD. Follow-up (censoring) for each patient will end at the earliest of the date of the practice's last CPRD data collection, the end of the patient's record collection (due to death or leaving the practice) and the date of the first record of statin/ antihypertensive (outcome for objective 3) or the date that therapy is discontinued (outcome for objective 4), or the end of the study period (31st December 2013).

Descriptive statistics will be used to describe the baseline demographics and cardiovascular risk distribution at index date (or closest recording) of the cancer survivors cross-tabulated with the non-cancer cohort for men and women separately and by age group , including frequency and percentages for categorical variables, and mean plus standard deviation for continuous variables. Monitoring of cardiovascular risk factors will be described for men and women separately and by age as proportions with a test in the past 1,3 and 5 years stratified by year since cancer diagnosis (or since one year prior to the index date for controls).
Robust standard errors will be used to deal with the fact that some patients will contribute person time to both the non-cancer and 1 year survivor cohorts, and to account for clustering at GP practice level.

Multivariable logistic regression modelling will be used to produce both unadjusted and adjusted ORs, to estimate the association between cancer survivorship and initiation of a statin/AHT in those with high CV risk, adjusted for relevant confounders/effect modifiers.

For the persistence analysis, we will measure time to event (discontinuation of statin/AHT) in cancer survivors versus non-cancer controls using Kaplein Meier curves. Summary statistics will be calculated for the baseline covariates for those patients who discontinued statins/AHT and for those who did not. Hazard ratios will be estimated for the associations between cancer survivorship compared with non-cancer controls and discontinuation of statin/AHT using the Cox proportional hazards model, controlling for confounding/interaction. Robust standard errors will be used for the above analyses to account for clustering at GP practice level in the final model.

Sample empty tables are provided in Appendices 6-8

Sensitivity analysis
1. For our main analysis, eligibility for a statin is based on having a recorded high risk score only. As a sensitivity analysis, the analysis for objective 3 will be repeated with a more comprehensive definition of high cardiovascular risk and therefore first eligibility for statin and/or AHT therapy based on the relevant guidelines that applied at the time to as follows:

Statins

* 2005 – 2007: CVD risk ≥20% over 10 years OR a read code in the clinical file for familial dyslipidaemia, OR elevated total cholesterol to high density lipoprotein (HDL) cholesterol ratio > 6.0, OR diabetes and age >40 years
* 2008-2013: Age >75, in addition to the above

AHT

* 2005 – 2011: CVD risk ≥20% over 10 years, AND Blood pressure equal to or over 140/90mmHg (stage 1 hypertension), OR Systolic BP>160 mm Hg / diastolic BP >100 mm Hg / (stage 2) severe hypertension
* 2011-2013: In addition to the above <80 years AND Stage 1 hypertension AND 1 or more of the following: target organ damage (heart/kidneys), established CVD, renal disease, diabetes

2. As stated in section H, we will repeat all analyses involving socioeconomic status among linked patients, accounting for patient-postcode based IMD, instead of practice-level IMD. This will allow for better control of IMD, at the expense of a smaller sample size.

M. Plan for addressing confounding

At the analysis stage we will assess for confounding by the covariates listed in Section K.

We will present the unadjusted odd ratios and 95% confidence intervals, overall and with stratification by covariates (gender, age, BMI, BP, cholesterol, cholesterol/HDL ratio, smoking status, alcohol status, SES, comorbidities (DM, CKD), cancer site, and time since index date). The association between cancer survivorship and uptake of preventive therapy will be adjusted for by each covariate in turn. If the adjusted OR differs to the unadjusted OR by around >10% we will consider the covariate a confounder to be included in the multivariate model.

The model will be built adding the confounders one by one, starting with the strongest. Once the full model is built we will then investigate whether any of the covariates modify the association between cancer and initiation of a statin/AHT by individually adding each factor to the model as an interaction term and performing a LRT. We will test for interaction with time since cancer diagnosis (<5/>5 years). We will report p values for the Likelihood Ratio Test for homogeneity and interpret for evidence of effect modification.

N. Plan for addressing missing data

Completeness of data for all variables will be reported, and quantifying completeness of blood pressure, lipids, CVD risk scores are part of Objective 1.

There may be missingness for covariates such as BMI, smoking, alcohol, HDL/cholesterol ratio. The main analyses will be conducted using data from individuals with complete data across the confounders to be included in the models. Comparison of variable distributions between individuals included in complete case analysis and those excluded due to missing data will be performed using Chi square tests, and t-tests as appropriate.

O. Limitations of the study design, data sources and analytical methods

Limitations in the context of this study include:
Confounding: while a range of co-variables will be included in the analysis, there may be some variables that we cannot account for as they have not been measured or are not available in CPRD eg. Family history of CVD, ethnicity, non-medical interventions such as diet and lifestyle.
Selection bias: The primary outcome is based on patients having a CVD risk score recorded in the CPRD dataset. There may be a substantial amount of missing data on CV risk scores if the doctor recorded the score in free text notes or never actually measured CVD risk score. We will compare the level of missingness between cancer survivors and non-cancer controls to assess whether missingness is associated with having cancer, and thus how much of an issue selection bias might be.

Misclassification: it is impossible to decipher whether a statin/AHT was offered and refused by the patient versus not offered at all when indicated. Misclassification is expected to be similar between exposed and controls (non-differential) and therefore might bias the result towards the null. We only have data on whether a patient was prescribed a therapy and not whether it was dispensed/taken by the patient. Over the counter (OTC) statin therapy is not captured in CPRD data and thus we might underestimate the uptake of statin therapy. However based on recent research on sales of OTC statins and consumer interviews, we don't expect this will be to a very large degree.[18]

P. Patient or user group involvement (if applicable)

At this stage we will not involve patient groups in the study, however we intend to approach relevant groups when preparing the manuscript for patient perspectives on the implication of the results.

Q. Plans for disseminating and communicating study results, including the presence or absence of any restrictions on the extent and timing of publication

We plan to publish the results of this study in a peer reviewed medical journal and to present results at national and international conferences. Components of this study, mainly related to the primary prevention of CVD with statins will be the subject of Kendal Chidwick's MSc Project Report.

Appendix 1

Table 1: Guidelines for the primary prevention of cardiovascular disease using statins and antihypertensive medicines (1998-2014)

Year published	Guideline	Indication for statin/AHT based on high CVD risk score	Indication for statin/AHT based on high risk (no CVD risk assessment required)
1998	JBS1: Joint British Societies' guidelines on prevention of cardiovascular disease in clinical practice	• As a minimum all individuals with an absolute CHD risk of 30% or more over 10 years.<br>• When it has been shown that those at highest risk have been effectively targeted the scientific evidence justifies a progressive expansion of coronary prevention from 30% down to 15% absolute CHD risk, linked to NHS resources needed to deliver effective preventive care.	Indication for AHT<br>• severe hypertension (systolic > 160 mm Hg and/or diastolic > 100 mm Hg)<br>Indication for statin<br>• familial hypercholesterolaemia or other inherited dyslipidaemia<br>• diabetes mellitus with associated target organ damage.
2000	National Service Framework. Chapter 2. Preventing coronary heart disease in high risk patients.	• CHD risk greater than 30% over ten years	
2004	NICE guidelines [CG18]. Essential hypertension: managing adult patients in primary care	Indication for AHT:<br>• Persistent blood pressure ≥140/90mmHg AND 10-year CVR score ≥20% OR 10-year CHD score ≥15%	Indication for AHT:<br>• Persistent high BP of ≥160/100mmHg<br>• Persistent blood pressure ≥140/90mmHg AND existing CVD or target organ damage
2005	JBS 2: Joint British Societies' guidelines on prevention of cardiovascular disease in clinical practice.	Indication for AHT:<br>• All those with CVD risk ≥20% over 10 years (i.e. high risk) AND<br>• Stage 1 hypertension (BP ≥140/90mmHg)<br><br>Indication for statin:<br>• All those with CVD risk ≥20% over 10 years (i.e. high risk).	Indication for AHT:<br>• elevated blood pressure > 160 mm Hg systolic or > 100 mm Hg diastolic, or lesser degrees of blood pressure elevation with target organ damage<br><br>Indication for statin:<br>• elevated total cholesterol to high density lipoprotein (HDL) cholesterol ratio > 6.0<br>• familial dyslipidaemia, such as familial hypercholesterolaemia or familial combined hyperlipidaemia.<br>• >40yo and type 1 or 2 DM
2005	World Health Organisation (WHO). Prevention of cardiovascular disease: guidelines for assessment and management of total cardiovascular risk.	10-year total CVD risk thresholds for intensive intervention:<br>• high-resource setting: 20%<br>• medium-resource setting: 30%<br>• low-resource setting: 40%	• total cholesterol ≥ 8 mmol/l (320 mg/dl) or low-density lipoprotein (LDL) cholesterol ≥ 6 mmol/l (240 mg/dl) or TC/HDL-C ratio > 8;<br>• persistent raised blood pressure (> 160–170/100–105 mmHg) (38–41, 43, 83);

			• type 1 or 2 diabetes, with overt nephropathy or other significant renal disease;<br>• patients with known renal failure or renal impairment.
2006	NICE guidelines [CG34] Hypertension: management of hypertension in adults in primary care.	Indication for AHT:<br>• Persistent blood pressure ≥140/90mmHg AND 10-year CVR score ≥20%	Indication for AHT:<br>• Persistent high BP of ≥160/100mmHg (any age)<br>• Persistent blood pressure ≥140/90mmHg AND existing CVD or target organ damage
2008	NICE guidelines [CG67] Lipid modification: Cardiovascular risk assessment and the modification of blood lipids for the primary and secondary prevention of cardiovascular disease	Indication for statin:<br>• Framingham score >20%	Indication for statin:<br>• Age 75+<br>• Familial hypercholesterolaemia<br>Use clinical assessment for people for whom an appropriate risk calculator is not available or appropriate[i]
2011	NICE guidelines [CG127] Hypertension in adults: diagnosis and management	Indication for AHT:<br>• Stage 1 hypertension (≥140/90mmHg) AND 10-year CVR score ≥20%	Indication for AHT:<br>• Stage 2 hypertension ≥160/100mmHg (any age)<br>• <80 years AND Stage 1 hypertension AND 1 or more of the following:<br>○ Target organ damage (heart/kidneys)<br>○ Established CVD<br>○ Renal disease<br>○ diabetes
2014	NICE guidelines [CG181] Cardiovascular disease: risk assessment and reduction, including lipid modification	Indication for statin[i]:<br>• QRisk2 score >10%	Indication for statin:<br>• Age 85+<br>• Type I diabetes and >40 years (regardless of CVD risk score)<br>• CKD (estimated glomerular filtration rate (eGFR) less than 60 ml/min/1.73m2 and/or albuminuria.

[i] Recognise that standard CVD risk scores will underestimate risk in people who have additional risk because of underlying medical conditions or treatments. These groups include:

- people treated for HIV
- people with serious mental health problems
- people taking medicines that can cause dyslipidaemia such as antipsychotic
- medication, corticosteroids or immunosuppressant drugs
- people with autoimmune disorders such as systemic lupus erythematosus, and other systemic inflammatory disorders.

			• Familial hypercholesterolaemia

1. JBS1: Joint British Societies' guidelines on prevention of cardiovascular disease in clinical practice. Heart 1998: 80(suppl. 2):S1-S29
2. 2000 National Service Framework. Chapter 2. Preventing coronary heart disease in high risk patients. Modern Standards and Service Models (2000).
3. NICE guidelines [CG18] Published date: 2004. Essential hypertension: managing adult patients in primary care
4. JBS 2: Joint British Societies' guidelines on prevention of cardiovascular disease in clinical practice. Heart 91(Suppl. 5), v1–v52 (2005).
5. World Health Organisation (WHO). Prevention of cardiovascular disease: guidelines for assessment and management of total cardiovascular risk.2005 http://www.who.int/cardiovascular_diseases/guidelines/Full%20text.pdf
6. NICE guidelines [CG34] Published date: June 2006. Hypertension: Management of hypertension in adults in primary care.
7. NICE guidelines [CG67] Published date: May 2008. Lipid modification: Cardiovascular risk assessment and the modification of blood lipids for the primary and secondary prevention of cardiovascular disease
8. NICE guidelines [CG127] Published date: August 2011. Hypertension in adults: diagnosis and management
9. NICE guidelines [CG181] Published date: July 2014. Cardiovascular disease: risk assessment and reduction, including lipid modification

*JBS = British Cardiac Society, British Hyperlipidaemia Association, British Hypertension Society, endorsed by the British Diabetic Association

Appendix 2
Figure A1– cancer validity work

Figure A1: Observed and expected incidence of common cancers comparing calculated incidences using our CPRD case definition with published Office for National Statistics incidences rates

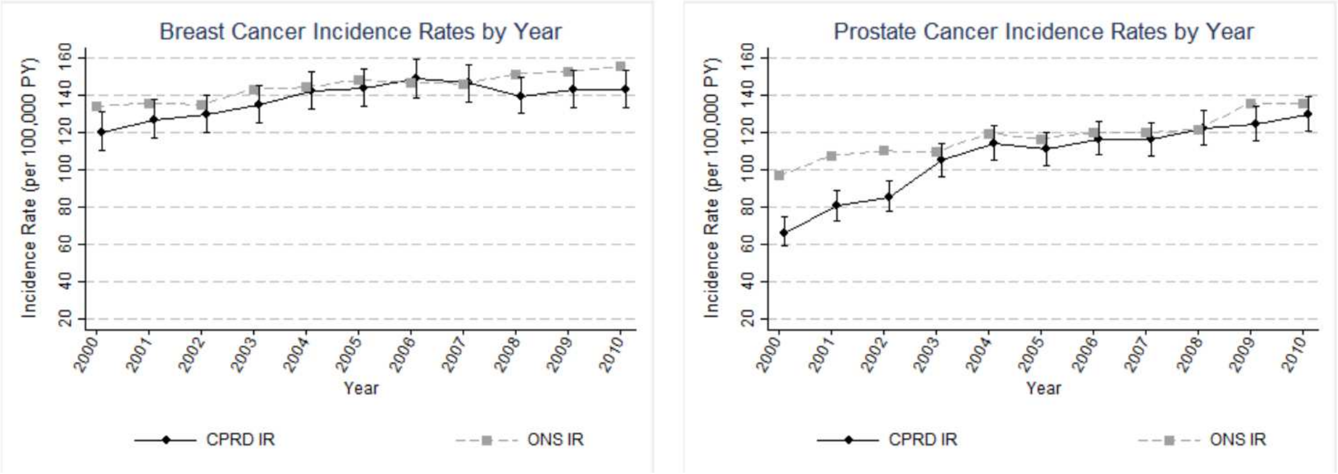

Appendix 3 – Method for identifying cancer cases in CPRD[i]

Webappendix Part 1 – additional methods detail and results

1) Identification of cancer cases in the Clinical Practice Research Datalink

To identify cancers in CPRD, the dictionary of Read codes (used by GPs to record clinical diagnoses) was systematically searched to find cancer-related codes using the keywords/word fragments below. The codes picked up by this search were then screened and those indicating malignancy were identified and classified by cancer type (done by KB, reviewed by LS). Each patient's record was then searched for these cancer codes. The earliest code for a particular cancer type was taken as the date of diagnosis. Only the first cancer per patient was considered; patients were then censored at this diagnosis for other cancers. This was due to the difficulty in distinguishing in the data between second de novo cancers and metastates.

Words and word fragments used to search Read code dictionary for cancer-related terms

MELANOMA NEOP TUMOUR CANCER MALIG CARCINOM LEUKAEM METASTA SARCOM LYMPHOM HODGKIN ACROSPIROMA ADAMANTINOMA ADENOACANTHOMA ADENOCARCIONOMA ADENOMA ADENOMATOSIS ANGIOENDOTHELIOMA ANGIOENDOTHELIOMATOSIS ANGIOMYXOMA APUDOMA ARGENTAFFINOMA ARYTHREMIA ASTROCYTOMA BLASTOM BOWEN BURKITT CARCINOID CHEMODECTOMA CHEMOTHERAPY CHLOROMA CHOLANGIOMA CHONDROMATOSIS CHORDOMA CRANIOPHARYNGIOMA CYSTADENOMA DESMOID ECCHONDROSIS EPENDYMOMA EPITHELIOMA ERYTHRAEMIA ERYTHRAEMIA ERYTHRAEMIA ERYTHREMIA ERYTHROPLASIA FIBROMA GAMMOPATHY GASTRINOMA GERMINOMA GLEASON GLIOMA GLUCAGONOMA HAEMANGIOENDOTHELIOMA HAEMANGIOENDOTHELIOMA HEMANGIOENDOTHELIOMA HEPATOMA HISTIOCYTIC HISTIOCYTOMA HISTIOCYTOSIS HYDATIDIFORM HYPERNEPHR HYPERNEPHR IMMUNOPROLIFERATIVE IMMUNOPROLIFERATIVE INSULINOMA KAHLER LEIOMYOMATOSIS LETTERER LYMPHANGIOMYOMATOSIS LYMPHOM LYMPHOPROLIFERATIVE MASTOCYTOMA MASTOCYTOSIS MECKEL MENINGIOMA MESENCHYMOMA MESONEPHROMA MESOTHELIOMA MESOTHELIOMA MYELODYSPLASTIC MYELOFIBROSIS MYELOMA MYELOMA MYELOPROLIFERATIVE MYELOSCLEROSIS MYELOSIS NEPHROMA NEURILEMMOMA NEURINOMATOSIS NEUROCYTOMA NEUROFIBROMATOSIS OSTEOCLASTOMA PAGET PANCOAST PANMYELOSIS PARAGANGLIOMA PERICYTOMA PINEALOMA PINEOCYTOMA PLASMACYTOMA PLASTICA POLYCYTHAEMIA POLYCYTHEMIA POLYEMBRYOMA PSEUDOMYXOMA RADIOTHERAPY SEMINOMA SEZARY TERATOMA TERATOMA THECOMA THROMBOCYTHAEMIA THROMBOCYTHEMIA THYMOMA VIPOMA WALDENSTROM [M] "ANGIOIMMUNOBLASTIC LYMPHADENOPATHY" "ATYPICAL FIBROXANTHOMA" BRILL CA CA-IN-SITU "DI GUGLIELMO" "GIANT PIGMENTED NAEVUS" "GIANT PIGMENTED NEVUS" "HEAVY CHAIN" "HUTCHINSON'S MELANOTIC" "MAST CELL" "MYCOSIS FUNGOIDES" "NEO/" "REFRACTORY ANAEMIA" "REFRACTORY ANEMIA" "RODENT ULCER" "STROMAL MYOSIS" "STRUMA OVARII" "TRANSITIONAL CELL PAPILLOMA, INVERTED" "UROTHELIAL PAPILLOMA"

---

[i] Supplement to: Bhaskaran K, Douglas I, Forbes H, et al. Body-mass index and risk of 22 specific cancers: a population-based cohort study of 5·24 million UK adults. *Lancet* 2014; published online Aug 14. http://dx.doi.org/10.1016/S0140-6736(14)60892-8.

Appendix 4 – Code list (CVD risk monitoring)

medcode	readterm	score_type	risk_level	readtermcat
7913	Coronary heart disease risk	Coronary risk score	No information from Read term	
10128	Cardiovascular event risk	Cardiovascular risk score	No information from Read term	
10302	Framingham coronary heart disease 10 year risk score	Coronary risk score	No information from Read term	
13283	Coronary heart disease risk	Coronary risk score	No information from Read term	
18581	Low risk of primary heart disease	Cardiovascular risk score	No information from Read term	
18948	Moderate risk of primary heart disease	Cardiovascular risk score	No information from Read term	
22210	High risk of primary heart disease	Cardiovascular risk score	No information from Read term	
24721	Framingham coronary heart disease 10 year risk score	Coronary risk score	No information from Read term	
26627	At risk of heart disease	Cardiovascular risk score	No information from Read term	
29433	High risk of heart disease	Cardiovascular risk score	No information from Read term	
36908	UKPDS 10yr coronary heart disease risk score	Coronary risk score	No information from Read term	
43934	Joint British Societies cardiac risk score	Cardiovascular risk score	No information from Read term	
43938	Framingham coronary heart disease 10 yr adjusted risk score	Coronary risk score	No information from Read term	
55103	JBS cardiovascular disease risk 10-20% over next 10 years	Cardiovascular risk score	Useful information in Read term	Oct-20
55104	JBS cardiovascular disease risk <10% over next 10 years	Cardiovascular risk score	Useful information in Read term	<10
55105	JBS cardiovascular disease risk >30% over next 10 years	Cardiovascular risk score	Useful information in Read term	>30
55109	JBS cardiovascular disease risk >20% up to 30% ov next 10 yr	Cardiovascular risk score	Useful information in Read term	>20 & <=30
71748	Coronary heart disease risk clinical management plan	Coronary risk score	No information from Read term	
85854	Review of patient at risk from coronary heart disease	Coronary risk score	No information from Read term	
95889	Assessing cardiovascular risk using SIGN score	Cardiovascular risk score	No information from Read term	
95948	QRISK cardiovascular disease 10 year risk score	Cardiovascular risk score	No information from Read term	
96275	ASSIGN score	Cardiovascular risk score	No information from Read term	
96886	Cardiovascular disease risk assessment done	Cardiovascular risk score	No information from Read term	
96899	Cardiovascular disease risk assessment indicated	Cardiovascular risk score	No information from Read term	
97641	Cardiovascular disease risk assessment	Cardiovascular risk score	No information from Read term	
98113	QRISK2 cardiovascular disease 10 year risk score	Cardiovascular risk score	No information from Read term	
98120	Framingham 1991 cardiovascular disease 10 year risk score	Cardiovascular risk score	No information from Read term	
98429	Cardiovascular disease high risk review	Cardiovascular risk score	No information from Read term	
100937	CVD (cardiovascular disease) risk assessment by third party	Cardiovascular risk score	No information from Read term	
101644	Consent given for cardiovascular health risk assessment	Cardiovascular risk score	No information from Read term	
104175	Joint British Societies cardiovascular disease risk score	Cardiovascular risk score	No information from Read term	
105223	At risk of cardiovascular disease	Cardiovascular risk score	No information from Read term	
105901	High risk of cardiovascular disease	Cardiovascular risk score	No information from Read term	

Appendix 5 – Code list (Statins)

prodcode	substance	prodcode	substance	prodcode	substance	prodcode	substance	prodcode	substance
47065	atorvastatin calcium	53813	cerivastatin sodium	9897	rosuvastatin calcium	58315	simvastatin	39652	simvastatin
47090	atorvastatin calcium	5009	cerivastatin sodium	17688	rosuvastatin calcium	45219	simvastatin	44650	simvastatin
47630	atorvastatin calcium	420	cerivastatin sodium	57763	rosuvastatin calcium	40601	simvastatin	51483	simvastatin
57348	atorvastatin calcium trihydrate	5251	cerivastatin sodium	57999	rosuvastatin calcium	53822	simvastatin	34481	simvastatin
28	atorvastatin calcium trihydrate	31658	cerivastatin sodium	15252	rosuvastatin calcium	49587	simvastatin	49062	simvastatin
56248	atorvastatin calcium trihydrate	58480	cerivastatin sodium	7554	rosuvastatin calcium	52625	simvastatin	51	simvastatin
75	atorvastatin calcium trihydrate	55207	cerivastatin sodium	9930	rosuvastatin calcium	39060	simvastatin	5148	simvastatin
55444	atorvastatin calcium trihydrate	5278	cerivastatin sodium	7347	rosuvastatin calcium	48867	simvastatin	10172	simvastatin/ezetimibe
51622	atorvastatin calcium trihydrate	18442	cerivastatin sodium	53460	rosuvastatin calcium	34381	simvastatin	17059	simvastatin/ezetimibe
58394	atorvastatin calcium trihydrate	9315	cerivastatin sodium	6213	rosuvastatin calcium	34316	simvastatin	7552	simvastatin/ezetimibe
51359	atorvastatin calcium trihydrate	4961	cerivastatin sodium	713	rosuvastatin calcium	32909	simvastatin	21020	simvastatin/ezetimibe
50236	atorvastatin calcium trihydrate	9316	cerivastatin sodium			13041	simvastatin	54606	
58110	atorvastatin calcium trihydrate	16186	ezetimibe/simvastatin	11815	simvastatin	53340	simvastatin	57329	
50272	atorvastatin calcium trihydrate	14219	ezetimibe/simvastatin	10206	simvastatin	50882	simvastatin	56165	
7374	atorvastatin calcium trihydrate	53770	fluvastatin sodium	10183	simvastatin	34376	simvastatin	54992	
52821	atorvastatin calcium trihydrate	2137	fluvastatin sodium	47948	simvastatin	45235	simvastatin	56065	
57117	atorvastatin calcium trihydrate	5985	fluvastatin sodium	56481	simvastatin	34891	simvastatin	56097	
55727	atorvastatin calcium trihydrate	9153	fluvastatin sodium	48018	simvastatin	52098	simvastatin	48973	
58418	atorvastatin calcium trihydrate	11627	fluvastatin sodium	34476	simvastatin	48078	simvastatin	56016	
52460	atorvastatin calcium trihydrate	8380	fluvastatin sodium	25	simvastatin	34969	simvastatin	48346	
53890	atorvastatin calcium trihydrate	379	fluvastatin sodium	39870	simvastatin	34955	simvastatin	48221	
2955	atorvastatin calcium trihydrate	1219	pravastatin sodium	51233	simvastatin	34907	simvastatin	58617	
17683	atorvastatin calcium trihydrate	54607	pravastatin sodium	57568	simvastatin	50754	simvastatin		
56841	atorvastatin calcium trihydrate	50925	pravastatin sodium	51166	simvastatin	44878	simvastatin		
52397	atorvastatin calcium trihydrate	51890	pravastatin sodium	51085	simvastatin	34746	simvastatin		
52398	atorvastatin calcium trihydrate	3690	pravastatin sodium	48431	simvastatin	50564	simvastatin		
49558	atorvastatin calcium trihydrate	54435	pravastatin sodium	34366	simvastatin	34535	simvastatin		
53594	atorvastatin calcium trihydrate	56893	pravastatin sodium	9920	simvastatin	34502	simvastatin		
3411	atorvastatin calcium trihydrate	490	pravastatin sodium	47774	simvastatin	54655	simvastatin		
47721	atorvastatin calcium trihydrate	40382	pravastatin sodium	50703	simvastatin	34545	simvastatin		
55034	atorvastatin calcium trihydrate	56607	pravastatin sodium	52257	simvastatin	46956	simvastatin		
52168	atorvastatin calcium trihydrate	56735	pravastatin sodium	52962	simvastatin	53908	simvastatin		
50788	atorvastatin calcium trihydrate	56916	pravastatin sodium	53087	simvastatin	33082	simvastatin		
53887	atorvastatin calcium trihydrate	1221	pravastatin sodium	56494	simvastatin	34879	simvastatin		

51134	atorvastatin calcium trihydrate	57397	pravastatin sodium	53415	simvastatin	7196	simvastatin
51200	atorvastatin calcium trihydrate	57108	pravastatin sodium	54240	simvastatin	52812	simvastatin
57834	atorvastatin calcium trihydrate	34820	pravastatin sodium	48058	simvastatin	22579	simvastatin
50963	atorvastatin calcium trihydrate	57296	pravastatin sodium	52676	simvastatin	44528	simvastatin
57836	atorvastatin calcium trihydrate	51676	pravastatin sodium	34353	simvastatin	34814	simvastatin
58041	atorvastatin calcium trihydrate	57137	pravastatin sodium	818	simvastatin	40340	simvastatin
51876	atorvastatin calcium trihydrate	1223	pravastatin sodium	54493	simvastatin	51715	simvastatin
54535	atorvastatin calcium trihydrate	43218	pravastatin sodium	39675	simvastatin	6168	simvastatin
52211	atorvastatin calcium trihydrate	730	pravastatin sodium	49061	simvastatin	46878	simvastatin
48518	atorvastatin calcium trihydrate	47988	pravastatin sodium	34560	simvastatin	42	simvastatin
53772	atorvastatin calcium trihydrate	52755	pravastatin sodium	53676	simvastatin	31930	simvastatin
745	atorvastatin calcium trihydrate	56146	pravastatin sodium	802	simvastatin	34312	simvastatin
56182	atorvastatin calcium trihydrate	55912	pravastatin sodium	54947	simvastatin	45245	simvastatin
5775	atorvastatin calcium trihydrate	32921	pravastatin sodium	54985	simvastatin	53966	simvastatin
49751	atorvastatin calcium trihydrate	36377	pravastatin sodium	55452	simvastatin	54819	simvastatin
50790	atorvastatin calcium trihydrate	48097	pravastatin sodium	50483	simvastatin	41657	simvastatin
52097	atorvastatin calcium trihydrate			54266	simvastatin	50670	simvastatin
56564	atorvastatin calcium trihydrate			54976	simvastatin	2718	simvastatin
52459	atorvastatin calcium trihydrate			48051	simvastatin	37434	simvastatin
55032	atorvastatin calcium trihydrate			52953	simvastatin	45346	simvastatin

Appendix 6

Table A1. Monitoring of blood pressure / lipid levels / CVD risk score: proportion with at least one test/score recorded in the past 1, 3 and 5 years in cancer survivors and controls, by time since diagnosis (or 1 year before index date for controls)

Men		<5 years since diagnosis		>5 years since Dx	
Age group	Test in the last 'x' years	Cancer N/D (%)	Controls N/D (%)	CANCER N/D (%)	CONTROLS N/D (%)
40-49	1 year				
	3 years				
	5 years	N/A	N/A		
50-59	1 year				
	3 years				
	5 years	N/A	N/A		
60-69	1 year				
	3 years				
	5 years	N/A	N/A		
70-79	1 year				
	3 years				
	5 years	N/A	N/A		
80+	1 year				
	3 years				
	5 years	N/A	N/A		

Appendix 7

Table A2. Overall cardiovascular risk distribution at index date (or closest recording)

	Males		Females	
Parameter at index date	Cancer n=	Controls n=	Cancer n=	Controls n=
Mean (SD) systolic blood pressure (mm Hg) (SD)				
40-49				
50-59				
60-69				
70-79				
80+				
Proportion (N/D, %) with systolic BP >160mm Hg				
40-49				
50-59				
60-69				
70-79				
80+				
Mean (SD) total cholesterol (mmol/l)				
40-49				
50-59				
60-69				
70-79				
80+				
Proportion (N/D, %) with total cholesterol > 8mmol/l				
40-49				
50-59				
60-69				
70-79				
80+				
Proportion (N/D, %) with a CVD risk score >20%				
All ages				
40-49				
50-59				
60-69				
70-79				
80+				

Appendix 8
Table A3. Proportion of patients with high CVD risk who were initiated on a statin / antihypertensive therapy (AHT) within 1 month of risk record: crude relative risk (OR: odds ratio) values for patients with cancer versus non-cancer controls

	Statin initiation				AHT initiation			
	Cancer	Non-cancer	Crude OR	Adjusted OR	Cancer	Non-cancer	Crude OR	Adjusted OR
	N/D (%)	N/D (%)	OR (95% CI)	OR (95% CI)	N/D (%)	N/D (%)	OR (95% CI)	OR (95% CI)
Entire cohort								
GENDER								
Males								
Females								
AGE								
40-45								
45-49								
50-54								
55-59								
60-64								
65-69								
70-74								
75-79								
80-85								
85+								
Time since index date*								
0-2 years								
2-4 years								
5+ (remission)								

**index date = 1 year after cancer diagnosis or date of joining the cohort for non-cancer controls*

R. References

1 Allemani C, Weir HK, Carreira H, Harewood R, Spika D, Wang XS, Bannon F, Ahn JV, Johnson CJ, Bonaventure A, Marcos-Gragera R, Stiller C, Azevedo ESG, Chen WQ, Ogunbiyi OJ, Rachet B, Soeberg MJ, You H, Matsuda T, Bielska-Lasota M, Storm H, Tucker TC, Coleman MP. Global surveillance of cancer survival 1995-2009: analysis of individual data for 25 676 887 patients from 279 population-based registries in 67 countries (CONCORD-2). Lancet. 2014.

2 *Quaresma M, Coleman MP, Rachet B. 40-year trends in an index of survival for all cancers combined and survival adjusted for age and sex for each cancer in England and Wales, 1971—2011: a population-based study. Lancet. 2014.*

3 National Cancer Survivorship Initiative: Department of Health, Macmillan Cancer Support, NHS Improvement. Living with and beyond cancer: taking action to improve outcomes (an update to the 2010 National Cancer Survivorship Initiative Vision). 2013 [cited December 2014]; Available from: www.ncsi.org.uk/wp-content/uploads/Living-with-and-beyond-2013.pdf

4 *Siegel R, Desantis C, Virgo K, Stein K, Mariotto A, Smith T, Cooper D, Gansler T, Lerro C, Fedewa S, Lin C, Leach C, Cannady RS, Cho H, Scoppa S, Hachey M, Kirch R, Jemal A, Ward E. Cancer treatment and survivorship statistics, 2012. CA Cancer J Clin. 2012.*

5 Oeffinger KC, Mertens AC, Sklar CA, Kawashima T, Hudson MM, Meadows AT, Friedman DL, Marina N, Hobbie W, Kadan-Lottick NS, Schwartz CL, Leisenring W, Robison LL, Childhood Cancer Survivor S. Chronic health conditions in adult survivors of childhood cancer. N Engl J Med. 2006; 355(15): 1572-82.

6 *Macmillan Cancer Support. Throwing light on the consequences of cancer and its treatment (report). 2013 [cited December 2014]; Available from: http://www.macmillan.org.uk/Cancerinformation/Livingwithandaftercancer/Lifeaftercancer/Long-term-effects-cancer.aspx*

7 Carver JR, Shapiro CL, Ng A, Jacobs L, Schwartz C, Virgo KS, Hagerty KL, Somerfield MR, Vaughn DJ, Panel ACSE. American Society of Clinical Oncology clinical evidence review on the ongoing care of adult cancer survivors: cardiac and pulmonary late effects. J Clin Oncol. 2007; 25(25): 3991-4008.

8 *Rowland JH, Bellizzi KM. Cancer Survivorship Issues: Life After Treatment and Implications for an Aging Population. J Clin Oncol. 2014.*

9 *Lenihan DJ, Cardinale D, Cipolla CM. The compelling need for a cardiology and oncology partnership and the birth of the International CardiOncology Society. Prog Cardiovasc Dis. 2010; 53(2): 88-93.*

10 Daher IN, Daigle TR, Bhatia N, Durand J-B.The Prevention of Cardiovascular Disease in Cancer Survivors. Tex Heart Inst J 2012;39(2):190-8)

11 *Organization WH, UNAIDS. Prevention of cardiovascular disease: World Health Organization; 2007.*

12 JBS 2: Joint British Societies' guidelines on prevention of cardiovascular disease in clinical practice. Heart 91(Suppl. 5), v1–v52 (2005).

13 World Health Organisation (WHO). Prevention of cardiovascular disease: guidelines for assessment and management of total cardiovascular. 2005 [cited May 2016]; http://www.who.int/cardiovascular_diseases/guidelines/Full%20text.pdf

14 NHS. Your questions on the NHS Health Check. 2014. [cited February 2016]; Available from: http://www.nhs.uk/Conditions/nhs-health-check/Pages/nhs-health-check-questions-faq.aspx

15 Van Staa T-P, Smeeth L, Ng ES-W, Goldacre B, Gulliford M. The efficiency of cardiovascular risk assessment: do the right patients get statin treatment? Heart Published Online First: 4 June 2013

16 Vinogradova Y, Coupland C, Brindle P et al. Discontinuation and restarting in patients on statin treatment: prospective open cohort study using a primary care database. BMJ 2016;353:i3305

17 Bhaskaran K, Douglas I, Forbes H, dos-Santos-Silva I, Leon DA, Smeeth L. Body-mass index and risk of 22 specific cancers: a population-based cohort study of 5.24 million UK adults. Lancet. 2014; 384(9945): 755-65.

18 Will CM, Weiner K.The drugs don't sell: DIY heart health and the over-the-counter statin experience. Soc Sci Med. 2015 Apr;131:280-8
